# Supplementary material for: Feasibility, safety and tolerability of estrogen and/or probiotics for improving vaginal health in Canadian African, Caribbean, and Black women: A pilot phase 1 clinical trial
Source: PLoS One. 2025 Jan 21;20(1):e0315576. doi: 10.1371/journal.pone.0315576 (PMC11750099; doi:10.1371/journal.pone.0315576)
Supplement: S1 File — (DOCX) [file pone.0315576.s002.docx]

**Feasibility, safety and tolerability of estrogen and/or probiotics for improving vaginal health in Canadian African, Caribbean, and Black women: A pilot phase 1 clinical trial**

**Supporting Information**

Biban Gill^1^, Jocelyn M. Wessels^1,2^, Christina L. Hayes^1^, Jenna Ratcliffe^1^, Junic Wokuri^3^, Elizabeth Ball^1^, Gregor Reid^4^, Rupert Kaul^5,6^, Jesleen Rana^3^, Muna Alkhaifi^3^, Wangari Tharao^3^, Fiona Smaill*^7^, & Charu Kaushic*^1^

^1^ McMaster Immunology Research Centre and Department of Medicine, McMaster University, Hamilton, ON, Canada.

^2^  Afynia Laboratories, Hamilton, ON, Canada.

^3^ Women’s Health in Women’s Hands Community Health Centre, Toronto, ON, Canada.

^4^ Departments of Microbiology & Immunology and Surgery, Western University, and Canadian Research and Development Centre for Human Microbiome and Probiotics, The Lawson Health Research Institute, London, ON, Canada.

^5^ Departments of Immunology and Medicine, University of Toronto, Toronto, ON, Canada.

^6^ Department of Medicine, University Health Network, Toronto, ON, Canada.

^7^ Department of Pathology and Molecular Medicine and Michael G. DeGroote Institute for Infectious Disease Research, McMaster University, Hamilton, ON, Canada.

* Corresponding authors

E-mail: kaushic@mcmaster.ca (CK)

E-mail: smaill@mcmaster.ca (FS)

**Table S1. Summary of intensity, duration and frequency of adverse events reported by participants**

**
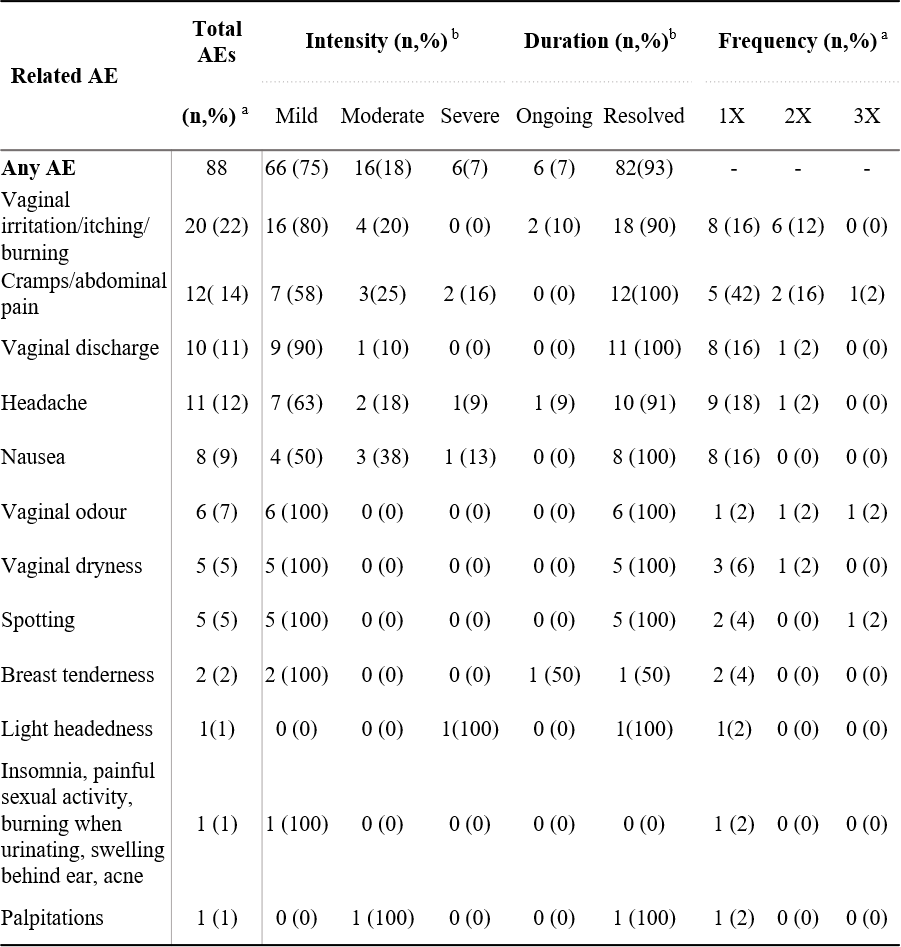
**

^a^Data shown as n (% total AEs or % of participants).^b^ Data shown as n % of the specific AEs).

**Table S2. Summary of adverse events and their reported relationship to intervention**


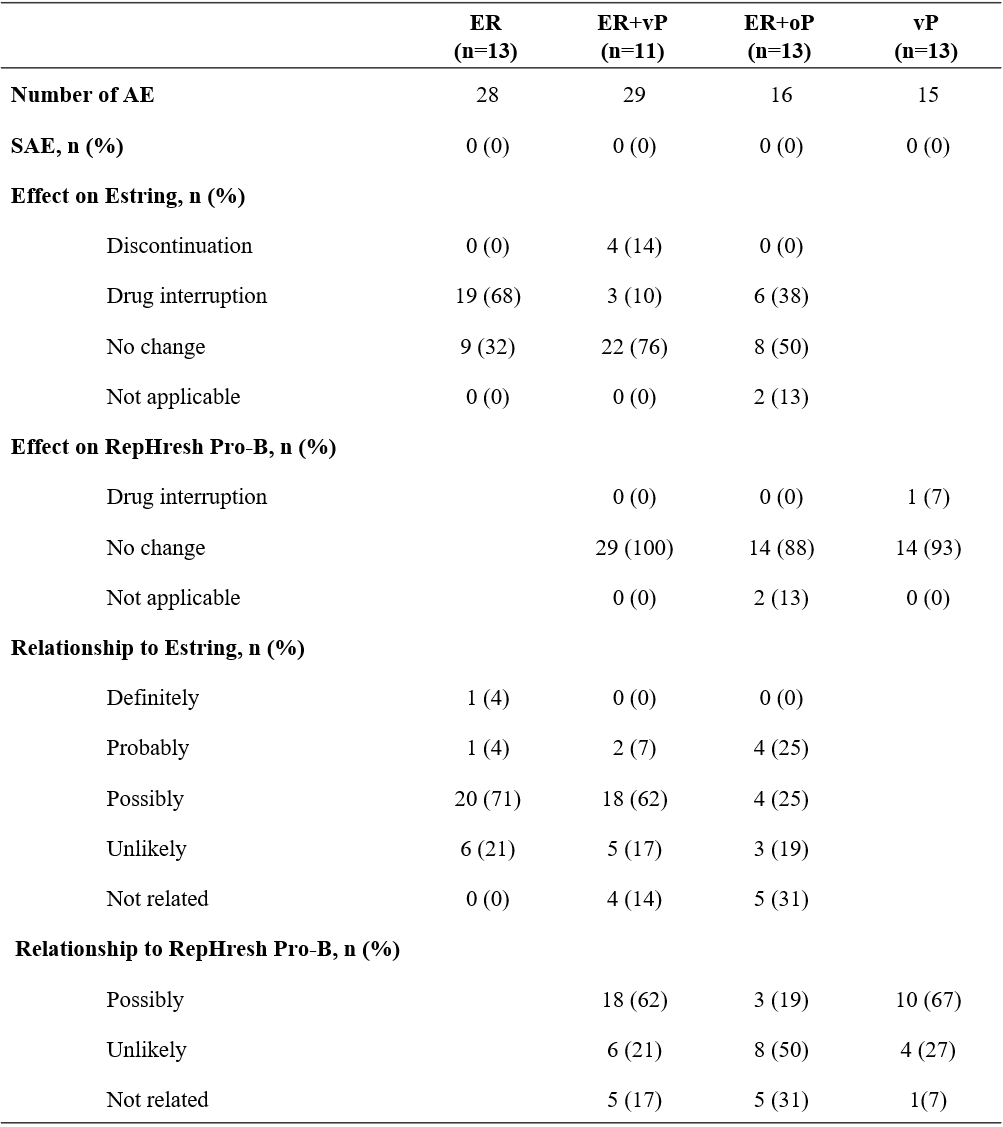


Data shown as n (% of AEs).

ER: Estring ; ER+vP : Estring and vaginal probiotic ; ER+oP Eststring and oral probiotic ; vP: vaginal probiotic
