# Supplementary material for: Feasibility, safety and tolerability of estrogen and/or probiotics for improving vaginal health in Canadian African, Caribbean, and Black women: A pilot phase 1 clinical trial
Source: PLoS One. 2025 Jan 21;20(1):e0315576. doi: 10.1371/journal.pone.0315576 (PMC11750099; doi:10.1371/journal.pone.0315576)
Supplement: S1 Protocol — (DOCX) [file pone.0315576.s003.docx]

| **FULL TITLE:** | Improving vaginal health to decrease biological risk of HIV-1 infection in Canadian ACB women: Estrogen and Probiotic Treatment for Vaginal Health |
| --- | --- |
| **Protocol No.** | CTN 308 |
| **Study Sponsor:** | McMaster University |
| **Principal Investigators:** | Dr. Charu Kaushic, PhD  Dr. Fiona Smaill, MSc, MBChB |
| **Study Support/ Funding:** | Canadian Institutes for Health Research, CIHR Canadian HIV Trials Network (CTN) |
| **ClinicalTrials.gov Identifier:** | NCT03837015 |
| **Version No.** | Version 1.4 |
| **Date:** | 24 May 2021 |

| **GCP Statement** |
| --- |
| This clinical study will be conducted in accordance with applicable Health Canada regulations, International Council for Harmonisation of Technical Requirements for Pharmaceuticals for Human Use (ICH) guidelines on current Good Clinical Practice (GCP), and the Declaration of Helsinki. |
|  |
| **Confidentiality Statement** |
| This clinical study protocol contains information which is of a confidential, trade-secret or proprietary nature. The protocol is for the use of McMaster University and their designated representatives participating in the investigational trial. It is not to be disclosed to any other person or party without the prior written approval of the principal investigators. |

Page 1 of 58

# TABLE OF CONTENTS

[TABLE OF CONTENTS 2](#_bookmark0)

[INVESTIGATOR AGREEMENT 6](#_bookmark1)

[STUDY CONTACT DETAILS 7](#_bookmark2)

[ABBREVIATIONS AND DEFINITONS 8](#_bookmark3)

[PROTOCOL SYNOPSIS 10](#_bookmark4)

[STUDY FLOW CHART 12](#_bookmark5)

1. [INTRODUCTION, BACKGROUND, AND STUDY RATIONALE 13](#_bookmark6)
   1. [POLYMICROBIAL VAGINAL MICROBIOME AND BACTERIAL VAGINOSIS BACKGROUND 14](#_bookmark7)
   2. [CURRENT TREATMENT OPTIONS 14](#_bookmark8)
   3. [SUMMARY OF NONCLINICAL AND CLINICAL DATA FOR THE STUDY TREATMENTS 14](#_bookmark9)
      1. [PREVIOUS STUDIES USING ESTRING 14](#_bookmark10)
      2. [PREVIOUS STUDIES USING PROBIOTICS (REPHRESH PRO-B) 17](#_bookmark11)
   4. [POTENTIAL RISKS AND BENEFITS TO HUMAN PARTICIPANTS 18](#_bookmark12)
   5. [STUDY RATIONALE 18](#_bookmark13)
2. [STUDY OBJECTIVES AND DESIGN 19](#_bookmark14)
   1. [OVERALL STUDY DESIGN 19](#_bookmark15)
   2. [PRIMARY OBJECTIVE(S) 19](#_bookmark16)
   3. [SECONDARY OBJECTIVE(S) 19](#_bookmark17)
   4. [EXPLORATORY OBJECTIVES(S) 20](#_bookmark18)
3. [SELECTION AND ENROLLMENT OF PARTICIPANTS 20](#_bookmark19)
   1. [NUMBER OF PARTICIPANTS 20](#_bookmark20)
   2. [INCLUSION CRITERIA 20](#_bookmark21)
   3. [EXCLUSION CRITERIA 20](#_bookmark22)
   4. [STRATEGIES FOR RECRUITMENT 22](#_bookmark23)
      1. INCENTIVE REFERRAL PROCESS 22
   5. [ENROLLMENT PROCEDURES 22](#_bookmark24)
4. [WITHDRAWAL OF PARTICIPANTS 23](#_bookmark25)
   1. [WITHDRAWAL CRITERIA 23](#_bookmark26)
   2. [PROCEDURES FOR DISCONTINUATION 23](#_bookmark27)
5. [RANDOMIZATION PROCEDURES 24](#_bookmark28)
   1. [RANDOMIZATION 24](#_bookmark29)
6. [STUDY TREATMENTS 24](#_bookmark30)
   1. [ESTRING 24](#_bookmark31)
      1. [PRODUCT DESCRIPTION 24](#_bookmark32)
      2. [PACKAGING, LABELING, STORAGE AND HANDLING 25](#_bookmark33)
      3. [EXPECTED SIDE EFFECTS 25](#_bookmark34)
   2. [REPHRESH PRO-B PROBIOTIC 26](#_bookmark35)
      1. [PRODUCT DESCRIPTION 26](#_bookmark36)
      2. [PACKAGING, LABELING, STORAGE AND HANDLING 26](#_bookmark37)
      3. [EXPECTED SIDE EFFECTS 26](#_bookmark38)
   3. [STUDY PRODUCTS, LABELING, SUPPLY AND ACCOUNTABILITY 27](#_bookmark39)
   4. [REGIMEN, ADMINISTRATION AND DURATION 27](#_bookmark40)
   5. [CONCOMITANT MEDICATIONS/NATURAL REMEDIES/FOODS 28](#_bookmark41)
   6. [CONCOMITANT ALCOHOL AND “STREET” DRUG USE 29](#_bookmark42)
   7. [PROHIBITED MEDICATIONS AND PROCEDURES 29](#_bookmark43)
   8. [PARTICIPANT ACCESS TO STUDY MEDICATION AT STUDY CLOSURE 29](#_bookmark44)
7. [RISKS AND PRECAUTIONS 29](#_bookmark45)
   1. [ACCEPTABLE METHODS OF BIRTH CONTROL 29](#_bookmark46)
   2. [MENTAL HEALTH SUPPORT 30](#_bookmark47)
   3. [RISK MANAGEMENT 30](#_bookmark48)
8. [CLINICAL AND LABORATORY EVALUATIONS 30](#_bookmark49)
   1. [CLINICAL EVALUATIONS 30](#_bookmark50)

[PELVIC EXAMINATIONS 30](#_bookmark51)

- 1. [LABORATORY EVALUATIONS AND SPECIMEN COLLECTION 30](#_bookmark52)
     1. [CLINICAL LABORATORY TESTS 31](#_bookmark53)
     2. [RESEARCH SAMPLE COLLECTION AND PROCESSING 31](#_bookmark54)
  2. [RESEARCH LABORATORY ASSAYS 34](#_bookmark55)
     1. [MICROBIOME ANALYSIS FROM CVL SAMPLES 34](#_bookmark56)
     2. [INNATE INFLAMMATORY CYTOKINE/CHEMOKINE ANALYSIS 34](#_bookmark57)
     3. [HIV TARGET CELL ASSAY 35](#_bookmark58)
     4. [IMMUNOLOGY PHENOTYPING, ETC ON PERIPHERAL BLOOD 35](#_bookmark59)
  3. [STORED RESEARCH SPECIMENS 35](#_bookmark60)
  4. [QUESTIONNAIRES 35](#_bookmark61)

1. [STUDY PROCEDURES 36](#_bookmark62)
   1. [SCHEDULE OF EVENTS 36](#_bookmark63)

[TABLE 4: SCHEDULE OF EVENTS 36](#_bookmark64)

- 1. [STUDY VISITS 37](#_bookmark65)
     1. [VISIT 1: SCREENING VISIT (DAY -30 TO -7) 37](#_bookmark66)
     2. [VISIT 2: BASELINE VISIT (DAY 0) 37](#_bookmark67)
     3. [VISIT 3 (DAY 14 ± 3 DAYS) – ON TREATMENT 38](#_bookmark68)
     4. [VISIT 4 (DAY 31 + 3 DAYS) – END OF TREATMENT 39](#_bookmark69)
     5. [VISIT 5 (DAY 37 ± 3 DAYS) – SAFETY FOLLOW-UP 39](#_bookmark70)

[IN-CLINIC VISIT (OPTIONAL) 40](#_bookmark71)

- 1. [EARLY TERMINATION VISIT 40](#_bookmark72)

[THE REASON FOR STUDY TERMINATION WILL BE RECORDED 40](#_bookmark73)

1. [EVALUATION, RECORDING, AND REPORTING OF ADVERSE EVENTS 41](#_bookmark74)
   1. [DEFINITIONS 41](#_bookmark75)
      1. [ADVERSE EVENT (AE) 41](#_bookmark76)
      2. [SERIOUS ADVERSE EVENTS (SAES) 41](#_bookmark77)
   2. [AE DESCRIPTIONS AND RECORDING 42](#_bookmark78)
      1. [INTENSITY 42](#_bookmark79)
      2. [RELATIONSHIP TO STUDY TREATMENT 42](#_bookmark80)
   3. [REPORTING AND EVALUATION OF SAES AND OTHER CLINICALLY SIGNIFICANT AES 43](#_bookmark81)
      1. [SAES 43](#_bookmark82)
      2. [OTHER CLINICALLY SIGNIFICANT AES (IF APPLICABLE) 44](#_bookmark83)
   4. [FOLLOW-UP FOR ADVERSE EVENTS 44](#_bookmark84)
   5. [PREGNANCY REPORTING 44](#_bookmark85)
   6. [STOPPING RULES 45](#_bookmark86)
2. [STATISTICAL CONSIDERATIONS 45](#_bookmark87)
   1. [GENERAL STUDY DESIGN 45](#_bookmark88)
   2. [SAMPLE SIZE CONSIDERATIONS/JUSTIFICATION 45](#_bookmark89)
   3. [DATA SETS TO BE ANALYZED 46](#_bookmark90)
   4. [ENDPOINTS/OUTCOME MEASURES 46](#_bookmark91)
   5. [ANALYSIS OF DEMOGRAPHIC AND BASELINE DATA 46](#_bookmark92)
   6. [ANALYSIS OF PRIMARY OUTCOME MEASURES 46](#_bookmark93)
   7. [ANALYSIS OF SECONDARY OUTCOME MEASURES 47](#_bookmark94)
3. [STUDY ETHICAL CONSIDERATIONS 47](#_bookmark95)
   1. [ETHICAL CONDUCT OF THE STUDY 47](#_bookmark96)
   2. [INFORMED CONSENT 47](#_bookmark97)
   3. [CONFIDENTIALITY 48](#_bookmark98)
   4. [RESEARCH ETHICS BOARD 48](#_bookmark99)
4. [GENERAL TRIAL CONDUCT CONSIDERATIONS 49](#_bookmark100)
   1. [ADHERENCE TO PROTOCOL 49](#_bookmark101)
      1. [PROTOCOL AMENDMENTS 49](#_bookmark102)
      2. [PROTOCOL DEVIATIONS 49](#_bookmark103)
   2. [MONITORING & AUDITING 49](#_bookmark104)
      1. [DATA SAFETY AND MONITORING COMMITTEE (DSMC) 49](#_bookmark105)
      2. [STUDY MONITORING 50](#_bookmark106)
      3. [EARLY TERMINATION OF THE TRIAL 50](#_bookmark107)
   3. [RECORD KEEPING 50](#_bookmark108)
      1. [DATA COLLECTION 50](#_bookmark109)
      2. [DATA CORRECTIONS 50](#_bookmark110)
      3. [SOURCE DOCUMENTS 50](#_bookmark111)
      4. [DATA MANAGEMENT 51](#_bookmark112)
      5. [RECORD RETENTION 51](#_bookmark113)
5. [DISCLOSURE AND PUBLICATION POLICY 51](#_bookmark114)
6. [REFERENCES 52](#_bookmark115)
7. [APPENDIX 58](#_bookmark178)
   1. [STUDY SEXUAL HISTORY QUESTIONNAIRE 58](#_bookmark179)

# INVESTIGATOR AGREEMENT

| **Protocol Title:** | **Improving vaginal health to decrease biological risk of HIV-**  **1 infection in Canadian ACB women: Estrogen and Probiotic Treatment for Vaginal Health** |
| --- | --- |
| **Protocol No.:** | CTN 308 |
| **Version No.:** | 1.4 |
| **Date:** | 24 May 2021 |

This clinical study will be conducted in accordance with applicable Health Canada regulations, ICH guidelines on current GCP, and the Declaration of Helsinki.

I confirm that I have read and understand this protocol and I agree to conduct this clinical study in accordance with the design and specific provisions of the protocol, with the exception of a change intended to eliminate an immediate hazard to participants. Any deviation from the study protocol will be documented in the case report form.

I agree to promptly report to the applicable ethics boards any changes in the research activity and all unanticipated problems involving risks to human participants or others. Additionally, I will not make any changes in the research without prior ethics and sponsor approval, except where necessary to ensure the safety of study participants.

| Name |  | Signature |  | Date (dd-mmm-yyyy) |
| --- | --- | --- | --- | --- |
| Name |  | Signature |  | Date (dd-mmm-yyyy) |

# STUDY CONTACT DETAILS

| **Role** | **Contact Details** |
| --- | --- |
| Medical Monitor | Dr. Mona Loutfy |
| SAE Reporting | Judy Needham  CIHR Canadian HIV Trials Network (CTN) |
| Study Support/Funding | CIHR Canadian HIV Trials Network (CTN) |
| Clinical Laboratory Facility | Toronto Public Health Ontario Laboratory |
| Specimen Analysis | Kaushic Lab McMaster Immunology Research Centre |
| Drug Supply | Bay Area Research Logistics |

ABBREVIATIONS AND DEFINITONS

| **Acronym / Abbreviation** | **Definition** |
| --- | --- |
| ACB | African, Caribbean and Black |
| ADR | Adverse Drug Reaction |
| AE | Adverse Event |
| BARL | Bay Area Research Logistics |
| BV | Bacterial vaginosis |
| CMC | Cervical mononuclear cells |
| COC | Combined oral contraceptives |
| CRF | Case Report Forms |
| CVL | Cervico-vaginal Lavage |
| DCs | Dendritic cells |
| DMPA | Depo-medroxyprogesterone Acetate |
| DSMC E2 | Data Safety and Monitoring Committee Estradiol |
| EIA | Enzyme immunoassay |
| FGT | Female genital tract |
| HC | Hormonal contraceptives |
| HDL | High-density lipoprotein |
| HIV | Human Immunodeficiency Virus |
| HSV | Herpes Simplex Virus |
| IUD | Intra-Uterine Device |
| LDL | Low-density lipoprotein |
| MIRC | McMaster Immunology Research Centre |
| NAAT | Nucleic Acid Amplification Test |
| P4 | Progesterone |
| PMBC | Peripheral blood mononuclear cell |
| PBS | Phosphate buffered saline |
| PSA | Prostate-specific antigen |
| REB | Research Ethics Board |
| SAE | Serious Adverse Event |
| SMC | Safety Monitoring Committee |

| **Acronym / Abbreviation** | **Definition** |
| --- | --- |
| STIs | Sexually Transmitted Infections |
| TPHL | Toronto Public Health Ontario Laboratory |
| WHIWH | Women’s Health in Women’s Hands |

# PROTOCOL SYNOPSIS

| **Full Title** | Improving vaginal health to decrease biological risk of HIV-1 infection in Canadian ACB women: Estrogen and Probiotic Treatment for Vaginal Health |
| --- | --- |
| **Short Title** | Estrogen and Probiotic Treatment for Vaginal Health |
| **Protocol and Version No.** | CTN 308, Version 1.3 |
| **Clinical Phase** | 1 |
| **Study Duration** | Enrollment period: August 2019 – December 2020 |
|  | Study period: August 2019 – March 2021 |
| **Sponsor** | McMaster University |
| **Number of Centres** | 1 |
| **Study Design** | A prospective, randomized, open-label, intervention study |
| **Primary Objective** | To determine the feasibility, safety and tolerability of administering low dose estrogen or probiotic, or in combination, to pre-menopausal women. |
| **Secondary Objectives** | 1. To determine if administration of local estrogen in combination with oral or vaginal probiotic treatment can enhance and stabilize *Lactobacillus* species in the vaginal tract. 2. To assess whether local estrogen in combination with oral or vaginal probiotic treatment can decrease innate inflammation in the cervico-vaginal secretions. 3. To determine if local estrogen in combination with oral or vaginal probiotic treatment decreases the number of HIV target cells in the genital tract. |
| **Exploratory Objectives** | 1. To determine if treatment effects persist one week after stopping treatment. |
| **Sample Size** | N = 80 |
| **Randomization** | 1:1:1:1 |
| **Study Population** | African, Caribbean and Black (ACB) pre-menopausal women, 18-49 yrs old |
| **Study Medication Description** | Estring® (estradiol vaginal ring) (DIN 02168898): Each ring contains 2 mg estradiol which is released slowly, 7.5 μg/24hours.  RepHresh™ Pro-B probiotic capsules (NPN [80012146](https://health-products.canada.ca/lnhpd-bdpsnh/info.do?licence=80012146)): One pill will be taken or administered orally or vaginally twice a day for 30 days. |
| **Administration and Dosing** | Participants in each group (n=20) will be randomized to receive treatment as follows:   - Group 1: Estring alone, - Group 2: Estring + RepHresh Pro-B capsules (administered vaginally) - Group 3: Estring + RepHresh Pro-B capsules (administered orally) |

|  | - Group 4: RepHresh Pro-B capsules (administered vaginally) alone |
| --- | --- |
| **Duration of Treatment** | 30 days |
| **Outcome Measures** | Primary:  The primary endpoints of this study are the feasibility of enrolling participants and the safety and tolerability of low dose estrogen treatment in combination with probiotics. Safety and tolerability will be evaluated by review of participant questionnaires, adverse events (AE) and serious adverse events (SAE). |
|  | Secondary:  The secondary endpoints of the study will be to observe changes in *Lactobacillus* species (enhanced proportion of lactobacilli) in the vaginal microbiota following Estring and oral/vaginal RepHresh treatment. Additional endpoints evaluated will include the change in inflammatory factors in the CVL and blood and HIV target T cells in the FGT, following intervention. |
| **Statistical Analysis** | Feasibility will be assessed by determining enrolment rate, retention rate, adherence to protocol and data completion rate. Tabulations and descriptive statistics will be employed in the analysis of all safety and tolerability observations.  Changes in microbiome and immunological outcome parameters, including cytokine levels, will be compared prior to and post treatment and among intervention groups with each participant acting as their own control. Statistical significance of changes in microbiome populations will be done by diversity metrics for each sample. Categorical variables will be compared among groups by Fisher’s Exact Test or Chi-Square. Continuous variables will be compared by student’s t-test,  Mann-Whitney Rank Sum Test, or by ANOVA. |

# STUDY FLOW CHART

**Participant Recruitment**

**On Treatment Visit**

**(Visit 3)**

- Physical Exam & Pelvic

Exam

Randomization/Enrollment Physical Exam & Pelvic Exam

- Sexual History Questionnaire
- Pregnancy Test
- Diary Review
- Dispense Study Products
- Assess AEs
- Sample Collection (cervical and blood)

•

•

**Baseline (Visit 2)**

Informed Consent Confirm Eligibility Physical Exam & Pelvic Exam

- Medical History
- Pregnancy Test
- STI Tests and safety bloods

•

•

•

**Screening (Visit 1)**

- Diary Review
- Dispense Study Products
- Assess AEs and Compliance
- Sample Collection (cervical)

**End of Treatment Visit (Visit 4)**

- Physical Exam & Pelvic Exam
- Diary Review and Return
- Return Study Products
- Assess AEs
- Safety bloods
- Sample Collection (cervical and blood)

**Safety Follow Up Visit (Visit 5)**

- Physical Exam & Pelvic Exam
- Diary Review and Return
- Assess AEs
- Sample Collection (if in-clinic)

**Data Analysis**

# INTRODUCTION, BACKGROUND, AND STUDY RATIONALE

Women are known to be at approximately 2-fold higher risk of Human Immunodeficiency Virus (HIV-1) acquisition than men. A number of biological factors are associated with increased risk in women, many of which are thought to enhance risk by increasing inflammation in the female genital tract (FGT) ([Ferreira et al., 2014](#_bookmark129)). Inflammation is known to increase HIV infection and replication through a number of mechanisms ([Ferreira et al., 2014](#_bookmark129)). There is extensive clinical and experimental evidence that susceptibility to infections and immune responses in the FGT are exquisitely regulated by the female sex hormones and their simulators such as hormonal contraceptives (HC) ([Kaushic et al., 2011](#_bookmark140); [Wira et al., 2015](#_bookmark176)). In general, progesterone (P4) and progestogens used in HC increase HIV susceptibility and inflammation while estradiol (E2) has been associated with decreased inflammation and thus increased protection ([Wira et al., 2015](#_bookmark176)). Estrogen is also known to enhance colonization by *Lactobacillus* species in the vaginal tract ([Mirmonsef et al., 2014](#_bookmark156)). A *Lactobacillus* dominant vaginal microbiome correlates with increased protection against HIV-1 and other sexually transmitted infections (STIs), while polymicrobial vaginal flora such as those seen in bacterial vaginosis (BV) is characterized by lack of *Lactobacillus* dominance and colonization by anaerobic bacteria ([Buve et al., 2014](#_bookmark121)). BV is also correlated with 2-fold increase in HIV-1 susceptibility and efforts to treat BV by antibiotics and/or probiotics have proven to be unsuccessful in establishing a *Lactobacillus* dominant “healthy” microbiome or preventing BV recurrence ([Martin et al., 1999](#_bookmark148)). About 40% of Black and Hispanic women have polymicrobial vaginal microbiomes, potentially putting them at a higher risk of HIV-1 infection ([Ravel et al.,](#_bookmark162) [2011b](#_bookmark162)). Here we are proposing an innovative approach to establishing a Lactobacillus- dominant vaginal microbiome in African, Caribbean, and Black (ACB) women who have polymicrobial microbiota and/or BV. We propose to achieve this by two means: (i) we will apply a low level of intra-vaginal estrogen, designed to increase vaginal receptivity for lactobacilli and (ii) administer probiotic bacteria, *Lactobacillus rhamnosus* GR-1 and *Lactobacillus reuteri* RC-14, as these species have been shown to disrupt BV biofilms, upregulate host immunity, improve vaginal barrier integrity and encourage recovery of a woman’s own endogenous lactobacilli ([Hummelen et al., 2010a](#_bookmark135); [Hummelen et al., 2010b](#_bookmark136)). This combined approach is hypothesized to confer multiple benefits not only to ACB women, who are higher risk of HIV, but to other women with recurrent BV as well. The estrogen-supported colonization with healthy lactobacilli will potentially displace BV-type anaerobes, enhance host immunity, normalize vaginal pH, and decrease FGT inflammation and clinical symptoms of BV. This should lead to an overall decrease in susceptibility to HIV-1 and other STIs in women. We have assembled a collaborative team of basic and clinical HIV researchers, along with community stakeholders and experts from outside of the HIV-1 field to test this innovative hypothesis. The knowledge generated from this project will provide new and valuable information regarding vaginal health and its role in protecting against STIs including HIV-1. The research results will be communicated through a series of planned knowledge translation activities to the community partners, ACB women and the general scientific community for widespread dissemination.

# Polymicrobial Vaginal Microbiome and Bacterial Vaginosis Background

Clinically, a polymicrobial vaginal flora may not result in a woman presenting with any symptoms. Similarly, BV can sometimes be asymptomatic. However, BV, typically described as a vaginal disease in which there is excessive growth of anaerobic bacteria, can be clinically diagnosed using the Amsel criteria. The Amsel criteria assess the presence of 3 out of 4 clinical signs of BV including a fishy odour, thin vaginal discharge, presence of clue cells during microscopy, and pH >4.5. In the laboratory, BV is assessed using the Nugent score, which determines the proportion of bacterial morphotypes present in a vaginal smear, following a Gram staining protocol. BV affects many women world-wide, however ACB women have an increased risk of BV as compared to other ethnicities, and the prevalence of BV is greatest in Africa ([Kenyon et al., 2013](#_bookmark142)). Similarly, 40% of Black and Hispanic women have polymicrobial vaginal microbiomes, while only 10% of Caucasian and Asian women have polymicrobial vaginal microbiomes ([Ravel et](#_bookmark161) [al., 2011a](#_bookmark161)), potentially putting ACB women at a higher risk of HIV-1 infection.

# Current Treatment Options

As most women with a polymicrobial vaginal microflora are asymptomatic, it is typically an incidental finding in research studies, and is not treated unless accompanied with clinical symptoms. Conversely, the preferred treatment for women presenting to their primary care physician with symptomatic BV is with oral or vaginal antibiotics/antiprotozoans including metronidazole or clindamycin ([Canada, 2013](#_bookmark122)). While these treatments are effective in temporarily eliminating the polymicrobial bacterial communities, it fails to establish a lactobacilli dominant vaginal microbial community, leading to recurrent episodes of BV. Thus, the major limitation of the current treatment regimens of BV is the high rate of disease recurrence, with 15-30% of women having a recurrence within 3 months ([Canada, 2013](#_bookmark122)).

# Summary of Nonclinical and Clinical Data for the Study Treatments

# Previous Studies using Estring

Estrogen-containing vaginal rings are commonly used in post-menopausal women during hormone replacement therapy or long-term treatment of urogenital disorders. **Table 1** lists the average concentration of estradiol found in the circulation or in the body tissues during several physiological stages and conditions in females. Conversely, **Table 2** lists the average concentration of estradiol found in the circulation following commonly employed estrogen therapies including Estring, NuvaRing, and oral contraceptive pills.

In post-menopausal women, use of Estring produces systemic levels of estradiol that are stable and maintained for an extended period of time ([Gabrielsson et al., 1996](#_bookmark130); [Naessen](#_bookmark159) [and Rodriguez-Macias, 2002](#_bookmark159); [Schmidt et al., 1994](#_bookmark167)). It is safe, highly effective, and generally well-accepted as a long-term treatment of disorders resulting from estrogen deficiency in post-menopausal women ([Barentsen et al., 1997](#_bookmark117); [Eriksen, 1999](#_bookmark128); [Henriksson](#_bookmark133)

[et al., 1996](#_bookmark133)). As Estring is associated with low systemic exposure to estradiol, it has a high safety margin because the risk of drug interactions is low ([Gabrielsson et al., 1996](#_bookmark130)). Use of Estring in post-menopausal women has been shown to lower vaginal pH to levels normally seen in cycling women, and induce maturation of the vaginal mucosa ([Barentsen](#_bookmark117) [et al., 1997](#_bookmark117); [Eriksen, 1999](#_bookmark128); [Henriksson et al., 1996](#_bookmark133)) without significantly changing endometrial thickness or uterine diameter ([Naessen and Rodriguez-Macias, 2002](#_bookmark159)). Estring has also been shown to prevent recurrence of urinary tract infections and decrease the number of recurrences per year in post-menopausal women, suggesting that local estrogen supplementation is capable of modifying the urogenital microbiome in a beneficial way ([Eriksen, 1999](#_bookmark128)). Although Estring is considered to have local effects, mainly on the vaginal and urogenital mucosae, its use in post-menopausal women has also been associated with improved serum lipid profiles including a reduction in serum low-density lipoprotein (LDL) cholesterol and LDL to high-density lipoprotein (HDL) profile, and an increase in serum HDL ([Naessen et al., 2001](#_bookmark160)). In addition to ease of use, post-menopausal women randomized to receive Estring to alleviate symptoms of urogenital estrogen deficiency were significantly less likely to have bleeding or spotting than women randomized to receive an estrogen-containing vaginal tablet ([Weisberg et](#_bookmark174) [al., 2005](#_bookmark174)).

While Estring is generally used as hormone replacement therapy in post-menopausal women, use of a continuous progesterone-containing intravaginal ring that was modelled after the Estring was well tolerated by pre-menopausal women undergoing cycles of in vitro fertilization ([Dragonas et al., 2007](#_bookmark127)). Although reports of Estring use in pre- menopausal women are lacking, the topical application of estrogens in doses higher than those contained in Estring have been successfully employed in the pediatric population to treat labial fusion ([Mayoglou et al., 2009](#_bookmark153); [Schober et al., 2006](#_bookmark168)). In one study 109 girls aged 0-36 months were treated with the topical estrogen-containing cream Premarin for a mean length of 3.7 months with minimal estrogen-related side effects (6 with minimal breast development, 1 with vaginal bleeding) which were resolved upon discontinuance of the treatment ([Schober et al., 2006](#_bookmark168)). Another study included girls up to age 14, and found topical application of estrogen cream for 1 month was a safe and effective treatment for labial fusion ([Kumetz et al., 2006](#_bookmark145)). A literature review of 6 observational studies on this topic found the side effects of topical estrogen use in the pediatric population to be mild and transient in nature, and included breast tenderness and changes in vulvar pigmentation ([Goldman, 2013](#_bookmark131)). Additionally, a review on the use of oral and transdermal estrogen therapies (including Premarin) for 1-6 months to treat failed pubertal induction and Turner syndrome in adolescents has been found to enhance systemic estradiol to normal pubertal levels ([Kenigsberg et al., 2013](#_bookmark141)).

In summary, intravaginal rings are well tolerated, and low-dose estrogen therapy is successfully used in the post-menopausal population, while slightly higher doses of topical and oral estrogens have been used in the pediatric and adolescent populations. We therefore expect the use of Estring in the pre-menopausal population to be acceptable by the study participants, and not associated with significant side effects.

**Table 1:** Average estrogen concentrations during different physiological stages and conditions in females.

| **Physiological**  **Condition** | **Average Concentration**  **of E2 (pg/mL)** | **Fluid or Tissue** | **Reference** |
| --- | --- | --- | --- |
| **Pre-pubertal Girls** | 2.6 (<0.5-17.7) | Serum | ([Courant et al.,](#_bookmark124) [2010](#_bookmark124)) |
| **During Puberty** | 19 (2.3-216.5) | Serum | ([Courant et al.,](#_bookmark124) [2010](#_bookmark124)) |
| **Pre-Menopausal Women (18-45 years)** | 61 (38-96) | Serum  (Day 3 of Menstrual Cycle) | ([Grisendi et al.,](#_bookmark132) [2014](#_bookmark132)) |
|  | 40 to 200-400 | Serum  (Over Menstrual Cycle) | [(Janfaza et al.,](#_bookmark137) [2006](#_bookmark137)) |
|  | 77.1 | Serum (Proliferative Phase) | ([Huhtinen et](#_bookmark134) [al., 2012](#_bookmark134)) |
|  | 157.3 | Serum (Secretory Phase) | ([Huhtinen et](#_bookmark134) [al., 2012](#_bookmark134)) |
|  | 535 | Endometrial Tissue (Proliferative Phase) | ([Huhtinen et](#_bookmark134) [al., 2012](#_bookmark134)) |
|  | 66 | Endometrial Tissue (Secretory Phase) | ([Huhtinen et](#_bookmark134) [al., 2012](#_bookmark134)) |
|  | 25 | Endometrial Tissue (Women on COCs) | ([Huhtinen et](#_bookmark134) [al., 2012](#_bookmark134)) |
| **During Pregnancy** | 350 | Serum | ([Tulchinsky et](#_bookmark172) [al., 1972](#_bookmark172)) |
| **Post-menopausal**  **women** | <20 | Serum | [(Janfaza et al.,](#_bookmark137) [2006](#_bookmark137)) |
|  | 9.8 (5.9-12.0) | Serum | [(Maruoka et al.,](#_bookmark150) [2014](#_bookmark150)) |
|  | 4.1 (15.0 pmol/L) | Serum | ([Naessen and](#_bookmark159)  [Rodriguez-](#_bookmark159) [Macias, 2002](#_bookmark159)) |
| **Post-menopausal women on Estring (12 months)** | 4.2 (15.4 pmol/L) Within normal post-  menopausal range and not significantly different from baseline values or untreated control women | Serum | ([Naessen and](#_bookmark159) [Rodriguez-](#_bookmark159) [Macias, 2002](#_bookmark159)) |

**Table 2:** Average concentration of estradiol found in the circulation following estrogen therapies

| **Estrogen Formulation** | **Route of Administrati**  **on** | **Administered in Formulation per**  **Day** | **Serum Concentration**  **of E2 (pg/mL)** | **Reference** |
| --- | --- | --- | --- | --- |
| **ESTRING** | Intravaginal Ring | 6.5-9.5μg | 4.2  (15.4 pmol/L) | ([Naessen and](#_bookmark159)  [Rodriguez-Macias,](#_bookmark159) [2002](#_bookmark159)) |
| **NuvaRing** | Intravaginal Ring | 0.12mg  (etonogestrel) | - | Nuvaring.com |
|  | 0.015mg  (ethinyl estradiol) | | - |  |
| **Premarin** | Vaginal Cream | 0.625mg | - | Premarin.com |
| **Estrance** | Intravaginal Tablet | 4mg (2mg/bid for 1 week in pre-menopausal  women) | 2344 | ([Tourgeman et al.,](#_bookmark171) [1999](#_bookmark171)) |
|  | Oral Tablet | 4mg (2mg/bid for 1 week in pre-menopausal  women) | 279 |  |
| **Oral Contraceptive**  **Pills** | Oral Tablet (Alesse) | 0.1mg  (levonorgestrel) | 77  (at steady state) | Pfizer.ca |
|  | 0.02mg (ethinyl estradiol) | |  |  |
|  | Oral Tablet (Lo Loestrin Fe-low dose pills) | 1mg (norethindrone  acetate) | - | Allergan.com |
|  |  | 0.01mg  (ethinyl estradiol) |  |  |
|  | Oral Tablet | 2mg norethindrone 100mu-g mestranol | 20-30 | ([Mishell et al.,](#_bookmark157) [1972](#_bookmark157)) |
|  | Oral Tablet | - | 2.5 | ([Huhtinen et al.,](#_bookmark134) [2012](#_bookmark134)) |

# Previous Studies using Probiotics (RepHresh Pro-B)

Previous studies using RepHresh Pro-B are summarized in the Investigator’s Brochure. Briefly, it is believed that RepHresh Pro-B helps maintain a healthy vaginal flora by reducing the number of pathogenic bacteria and yeast on vaginal surfaces. Further, the probiotics contained within RepHresh Pro-B (RC-14 and GR-1) are thought to confer vaginal health benefits and can be used in conjunction with antibiotics to help treat bacterial vaginosis (BV). The mechanism of action of orally administered RepHresh Pro- B and RC-14/GR-1 includes modulation of the vaginal microenvironment, prevention of pathogenic bacterial growth, and may include the modification of certain immune parameters in the systemic circulation. The mechanism of action of vaginally administered RepHresh Pro-B and RC-14/GR-1 includes host immunomodulation, enhanced vaginal barrier function, and increased vaginal lactate.

According to the available literature, there have not been any reports of overdose or toxic effects due to RepHresh Pro-B when 1 or 2 capsules are administered daily, and these studies often included fragile patient populations (spinal cord injury, post-menopausal women, pregnant women, cervical cancer patients, women positive for Human Immunodeficiency Virus (HIV)). The most common side effects of RepHresh Pro-B reported in the literature, which were rare and similar to those reported in placebo groups, were abdominal discomfort, vomiting, or vaginal discomfort. Thus, the literature indicates that oral and vaginal administration of the probiotics contained within RepHresh Pro-B (RC-14 and GR-1) can improve vaginal health, are safe, and well tolerated even in fragile patient populations.

# Potential Risks and Benefits to Human Participants

There can be bruising associated with blood collection and very rarely infection. There can be discomfort associated with the vaginal examination and collection of samples. Participants will have an HIV test and testing for STIs (Chlamydia and gonorrhea); learning about a positive result can be associated with distress, but appropriate counseling and referral for care will be provided.

Based on the present body of published literature we anticipate the use of Estring and RepHresh in the pre-menopausal population will be acceptable to the study participants, and not associated with significant side effects. The potential benefits to study participants might include but not be limited to: improved vaginal health (decreased polymicrobial flora and urinary tract infections, enhanced vaginal lubrication) ([Bisanz et al., 2014](#_bookmark119); [Kruse et](#_bookmark143) [al., 2010](#_bookmark143); [Kruse et al., 2009](#_bookmark144)), long term vaginal colonization by Lactobacilli, decreased vaginal inflammation, enhanced serum lipid profiles ([Naessen et al., 2001](#_bookmark160)), and favourable effects on bone density and health ([Kruse et al., 2010](#_bookmark143)). Although we do not anticipate any major risks, the potential risks to study participants include risks described in any woman on estrogen therapies, as listed in section 10 (Evaluation, Recording, and Reporting of Adverse Events).

RepHresh should not be used if nausea, fever, vomiting, bloody diarrhea or severe abdominal pain occur, nor in the setting of an immunocompromising condition (e.g. AIDS, lymphoma, patients undergoing long-term corticosteroid treatment). Its use should be discontinued if symptoms of digestive upset (e.g. diarrhea) occur, worsen or persist beyond 3 days or if an unfamiliar odour, discharge, pain or other symptoms of concern occur.

# Study Rationale

A large body of data exists demonstrating that female sex hormones regulate susceptibility and immune responses in the FGT (reviewed in ([Kaushic et al., 2011](#_bookmark140))). While P4 is known to enhance susceptibility and induce inflammatory responses following viral infection in animal models, E2 decreases susceptibility ([Wira et al., 2015](#_bookmark176)). Estrogen

also directly influences the microbiota in the FGT. *Lactobacillus* species are the dominant species in the healthy vaginal microbiota, whereas polymicrobial colonization dominated by anaerobic bacterial species is correlated with BV, a common inflammatory condition that enhances HIV-1 infection ([Brotman et al., 2014](#_bookmark120)). Estrogen is known to increase production of glycogen in vaginal epithelial cells, a substrate for *Lactobacillus* species*,* thereby enhancing colonization by lactobacilli ([Mirmonsef et al., 2014](#_bookmark156)). Clinical studies show that oral administration of probiotic *Lactobacillus* can facilitate replacement of the BV microbiota with a *Lactobacillus* dominated microbiota in the vaginal tract ([Mastromarino et al., 2013](#_bookmark151); [Reid, 2008](#_bookmark163); [Reid et al., 2009](#_bookmark164)). A healthy microbiota has beneficial effects on the immune system ([Buve et al., 2014](#_bookmark121); [Karimi et al., 2009](#_bookmark139)). However, the shift from a BV type flora is usually temporary and most women experience a recurrence of BV. Delivering local estrogen might help in establishing colonization by lactobacilli and maintaining a *Lactobacillus* dominant vaginal microbiome. This in turn might diminish inflammation and HIV target cells in the vaginal mucosa.

The study is designed as a proof of concept with 4 study arms in order to evaluate the safety and feasibility of the use of Estring and RepHresh by comparing the combination of Estring with RepHresh to single treatment with either EString or RepHresh. As well oral administration of RepHresh with Estring will be compared with vaginal administration of RepHresh with Estring to evaluate the different route of administration of RepHresh on outcomes. Oral RepHresh alone is not included since this has been tested in previous studies (Vujic et al., 2013). The study is designed to compare outcomes in each woman prior to and post-treatment, with each woman serving as her own control.

# STUDY OBJECTIVES AND DESIGN

# Overall Study Design

This prospective, randomized, open-label intervention pilot study will enroll a cohort of 80 African, Caribbean and Black (ACB) women into 4 groups. Participants in each group (n=20) will be randomized to receive treatment with Group 1: Estring alone, Group 2: Estring + RepHresh Pro-B capsules (administered vaginally), Group 3: Estring + RepHresh Pro-B capsules (administered orally), or Group 4: RepHresh Pro-B capsules (administered vaginally). The treatment duration will be 30 days with a 7-day follow up period to examine how long the treatment effect lasts.

# Primary Objective(s)

1. To determine the feasibility, safety and tolerability of administering low dose estrogen or probiotic, or in combination, to pre-menopausal women.

# Secondary Objective(s)

1. To determine if administration of local estrogen in combination with oral or vaginal probiotic treatment can enhance and stabilize *Lactobacillus* species in the vaginal tract.
2. To assess whether local estrogen in combination with oral or vaginal probiotic treatment can decrease innate inflammation in the cervico-vaginal secretions.
3. To determine if local estrogen in combination with oral or vaginal probiotic treatment decreases the number of HIV target cells in the genital tract.

# Exploratory Objectives(s)

1. To determine if treatment effects persist one week after stopping treatment.

# SELECTION AND ENROLLMENT OF PARTICIPANTS

# Number of Participants

Eighty (80) participants will be enrolled at one study site located in Toronto, Ontario, Canada.

# Inclusion Criteria

Participants will be eligible for the study if they meet the following criteria:

1. Women 18-49 years of age, inclusive
2. African, Caribbean, Black
3. Pre-menopausal women in good general health, as determined by the investigator
4. Uterus and cervix present
5. Negative pregnancy test
6. Currently practicing barrier or non-hormonal forms of contraception, and planning to continue, for the duration of the study (barrier contraceptive, abstinence)
7. Willing to undergo a pelvic exam by a female nurse/female doctor
8. Willing to abstain from vaginal intercourse for 48 hours prior to sampling, over the entire course of the study
9. Able to understand, comply and consent to protocol requirements and instructions
10. Able to attend scheduled study visits and complete required investigations

# Exclusion Criteria

Participants who meet any of the following criteria are not eligible for admission to the study:

1. Currently lactating
2. Pregnant: suspected, current or in the last 12 months
3. Irregular menstrual cycle (less than 6 periods in a year) not related to contraceptive use, pregnancy or breastfeeding
4. Post-menopausal
5. Hormonal Contraceptive use or other hormonal treatment in the past 3 months
6. Current Intra-Uterine Device (IUD) use
7. Positive test result for Gonorrhea and/or Chlamydia
8. Clinically obvious genital ulceration/lesions
9. Symptomatic vaginal yeast infection or clinically significant vaginal discharge
10. HIV-positive
11. Any clinically significant abnormality on screening safety blood tests, that in the opinion of the investigator would preclude enrolment.
12. Diagnosed blood clotting disorder (e.g. venous thromboembolism such as deep vein thrombosis or pulmonary embolism, or active thrombophlebitis), including known thrombophilic disorders (e.g. protein C, protein S, or antithrombin deficiency)
13. Any genital tract procedure (e.g. biopsy) within the past 6 months
14. Use of oral probiotic supplement, oral antibiotics or oral steroids within the past 30 days of the Baseline Visit (Visit 2).
15. Current use of any vaginal products (except tampons) such as spermicides, microbicides, douching or drying products, antifungals, or steroids. Douching products include any fluid used to irrigate/rinse out the vagina, including solutions such as vinegar and water or pre-packaged commercial products labeled for vaginal use. Participants will be eligible for enrolment if they have discontinued use of any vaginal products at least 48 hours prior to the baseline visit and agree not to use such products for the duration of the study.
16. Known intolerance of Lactobacillus-containing probiotic supplements
17. Undiagnosed abnormal genital bleeding
18. Known, suspected, or history of breast cancer
19. Known or suspected estrogen-dependent malignant neoplasia (e.g. endometrial cancer)
20. Currently taking immunosuppressive drugs such as (Heazathioprine (Imuran), basiliximab (Simulect), cyclosporine (Neoral, Sandimmune), daclizumalthab (Zenapax), muromonab-CD3 (OKT3, Orthoclone OKT3), mycophenolate (CellCept), tacrolimus (FK506, Prograf), sirolimus (Rapamune), prednisone (Deltasone, Orasone), or oral corticosteroids (glucocorticoids)
21. Known or suspected hypersensitivity to any component of the Estring or RepHresh Pro-B products
    1. ESTRING: 17 β-estradiol, silicone elastomer, barium sulphate, silicone fluid
    2. RepHresh Pro-B: Lactobacillus reuteri RC-14 (ATCC55845), Lactobacillus rhamnosus GR-1 (ATCC55826), Anhydrous dextrose, potato starch, microcrystalline cellulose, magnesium stearate, gelatin, titanium dioxide
22. Diagnosis of endometrial hyperplasia
23. Known liver dysfunction or disease; as long as liver function tests have failed to return to normal
24. Active or past history of arterial thromboembolic disease (e.g. stroke, myocardial infarction, coronary heart disease)
25. Partial or complete loss of vision due to ophthalmic vascular disease
26. Porphyria
27. Concomitant medication which in the opinion of the investigator may be associated with a significant drug interaction with the estrogen in Estring.
28. The conditions below are grounds for exclusion based on the opinion of the investigator:
    1. Risk factors for estrogen dependent tumours, e.g. first degree heredity for breast cancer
    2. Diabetes mellitus with or without vascular involvement
    3. Migraine or (severe) headache
    4. Epilepsy
    5. A history of, or risk factors for, thromboembolic disorders
    6. Systemic lupus erythematosus
    7. Otosclerosis
    8. Cholelithaiasis
    9. Leiomyoma (uterine fibroids)
    10. Endometriosis
    11. A history of endometrial hyperplasia
    12. Hypertension
    13. Asthma
    14. Diagnosed anemia

# Strategies for Recruitment

The study will be advertised within the Women’s Health and Women’s Hands Clinic in Toronto as well as at various Greater Toronto Area community organizations (contingent on their permission) through flyers, recruitment cards, and by encouraging service providers and staff at these organizations to let their clients know about the study. The study will also be advertised on social media, both through the WHIWH pages as well as paid advertisements. All recruiting materials will be approved by the Institutional REB. Interested participants will be instructed to phone the research nurse to learn more about the study.

- - 1. **Incentive Referral Process**: Participants who successfully completed the study will be contacted by phone by the research nurse and offered $25 if they refer a friend to participate. The process for this would be that the friend would be given the research nurse's contact information by the former participant and when they call to set up a screening visit, the friend will provide the name of their referral. That person will then receive the $25 (through email transfer or in person at the clinic) once the friend attends their scheduled screening visit. This compensation is related to attending the screening visit only and is not contingent upon enrollment (attending baseline visit – Visit 2) in the study.

# Enrollment Procedures

Prior to study enrollment, all prospective participants will have a telephone conversation with the study research nurse where they will be provided with the relevant study specific information. The research nurse will provide information about the study and procedures that will be performed during the study and ask them about their general health. The research nurse will review inclusion and exclusion criteria for each potential participant by phone. Participants will be informed that they can withdraw at any time, and that the study is completely confidential. If they are interested in participating in the study, they

will schedule an in-clinic visit for more detailed discussion and screening. At this time, the research nurse will make sure that the study participant understands the study, including risks, benefits, and procedures. Only those willing to sign the informed consent forms will proceed to the screening, and potential enrollment.

# WITHDRAWAL OF PARTICIPANTS

# Withdrawal criteria

All participants are free to withdraw from participating in this study at any time and for whatever reason, specified or unspecified, and without prejudice to his or her medical care by a physician.

Reasons for participant withdrawal may include, but are not limited to, the following:

- For non-adherence with study visits or non-compliance with treatment interventions
- If it is in the participant’s best interest according to the Investigator’s clinical judgement
- Safety reasons (e.g. Serious adverse drug reactions, potential significant drug interaction)
- Participant’s request
- Sponsor’s request
- Pregnancy
- Participant is lost to follow-up. The Investigator will attempt to reach the participant before considering the participant lost-to-follow-up. These actions will be reported on the appropriate page of the CRF and in the participant’s file.

# Procedures for Discontinuation

If a participant withdraws or is removed from the study for any reason prior to the completion of the study, the reason for and the date of discontinuation, and date of the last dose of the study medication, must be recorded in the appropriate section of the CRF.

At the time of discontinuation, every effort should be made to ensure that:

- - 1. Procedures and evaluations scheduled for the Final Study Visit are performed, including assessments of AEs and medication compliance
    2. Study treatment compliance is performed
    3. Study treatments are returned and inventoried
    4. Study staff make an appointment for safety follow-up visit(s) if required

All premature discontinuations and their cause must be documented by the Investigator on the appropriate CRF pages, e.g. *Final Subject Status, Adverse Events, Participants not completing the study* should be fully evaluated (i.e., Final Visit procedures performed), wherever possible.

Any participants who discontinue participation due to an unresolved clinically significant AE will be followed until satisfactory clinical resolution is achieved and the AE documented in the CRF regardless of disease severity. For all AEs that require the participant to be discontinued from the study, relevant clinical assessments and laboratory tests will be repeated at least on a monthly basis until final resolution or stabilization of the event(s). These visits should be entered into the CRF as unscheduled Visits. If a participant is withdrawn from the study due to study treatment intolerability, an AE should be recorded **regardless of grade.**

# RANDOMIZATION PROCEDURES

# Randomization

Participants will be randomized to one of four treatment arms in a 1:1:1:1 ratio in an open- label manner. The treatment arms are:

- - - - - Group 1: Estring alone
        - Group 2: Estring + RepHresh Pro-B capsules (administered vaginally)
        - Group 3: Estring + RepHresh Pro-B capsules (administered orally)
        - Group 4: RepHresh Pro-B capsules (administered vaginally)

Prior to study commencement Bay Area Research Logistics (BARL) will develop a randomization scheme in blocks of random size 4 and 8 using an electronically generated randomization code ([www.randomization.com](http://www.randomization.com/)) and prepare identically appearing sequentially numbered study kits. The randomization code will be stored by BARL and sent to the investigators at the end of the study, after the database has been frozen.

At the baseline visit, the study nurse will dispense the next sequentially numbered study kit containing one of the treatments listed above, and only once the kit has been opened, will the participant and study nurse/doctor know which arm of the study the participant has been randomly assigned. This information will be provided with any SAE information to the medical monitor for review.

# STUDY TREATMENTS

# ESTRING

# Product Description

Generic name: 17 β-Estradiol

Brand Name: ESTRING Vaginal Ring, 2 mg (DIN 02168898)

Estring (estradiol vaginal ring) is a slightly opaque ring, made of a silicone elastomer sheath surrounding a whitish silicone elastomer core, containing a drug reservoir of 2 mg estradiol, barium sulphate as a marker and silicone fluid as a dispersing agent. Each ring contains 2 mg estradiol which is released slowly, 7.5 μg/24hours. The ring has an outer diameter of 55 mm with a cross-section diameter of 9.5 mm.

# Packaging, Labeling, Storage and Handling

Each Estring ring is individually packaged in a heat-sealed rectangular pouch consisting of, from outside to inside: Polyester/aluminum foil/low density polyethylene. The pouch is provided with a tear-off notch on one side. Each pouch is packed into a cardboard carton containing a Patient Information Leaflet. Each Estring ring package will be labeled as an investigational product in accordance with Health Canada regulations, as per section 6.3 below.

Estring should be stored at room temperature (15-30oC) and kept out of reach of children and pets.

# Expected Side Effects

The most frequently reported side effect is increased vaginal secretions. Many of these vaginal secretions are like those that occur normally, and indicates that the Estring is working. Vaginal secretions that are associated with a bad odour, vaginal itching or other signs of vaginal infection are NOT normal and may indicate a risk or cause for concern. Other side effects may include vaginal discomfort, abdominal pain, or urogenital itching.

The following adverse events were seen in studies with Estring:

- - - - Vaginal bleeding/spotting (4%)
      - Headache (13%)
      - Breast tenderness (1%)
      - Leg edema (swelling (1-3%)

Possible serious side effects of estrogen use include:

- - - - Heart attack
      - Stroke
      - Blood clots
      - Dementia
      - Breast cancer
      - Cancer of the lining of the uterus (womb)
      - Cancer of the ovary
      - High blood pressure
      - High blood sugar
      - Gallbladder disease
      - Liver problems
      - Changes in your thyroid hormone levels
      - Enlargement of benign tumours of the uterus (“fibroids”)

Less serious, but common side effects include:

- - - - Headache
      - Breast pain
      - Irregular vaginal bleeding or spotting
      - Stomach or abdominal cramps, bloating
      - Nausea and vomiting
      - Fluid retention
      - Vaginal yeast infection

# REPHRESH PRO-B PROBIOTIC

# Product Description

Brand Name: RepHresh Pro-B (NPN 80012146)

RepHresh Pro-B is a patented probiotic feminine supplement containing 2.5 billion CFU of both Lactobacillus reuteri RC-14 and Lactobacillus rhamnosus GR-1. Lactobacillus reuteri RC-14 (ATCC55845) originates from the vagina of a healthy woman. Lactobacillus rhamnosus GR-1 (ATCC55826) originates from the distal urethra of a healthy women. Each capsule contains non-medicinal ingredients of two fillers of anhydrous dextrose and potato starch as well as microcrystalline cellulose as a binder. The capsules are composed of gelatin and titanium dioxide. Magnesium stearate is a lubricant for the capsule.

# Packaging, Labeling, Storage and Handling

RepHresh Pro-B is packaged in 30 capsule containers commercially, and each study kit requiring RepHresh Pro-B will be prepared by BARL and contain 2 commercial containers of 30 capsules each, for a total of 60 capsules. RepHresh Pro-B will be packaged, and labeled as investigational product in accordance with Health Canada regulations, as per section 6.3 below.

RepHresh Pro-B should be stored at room temperature (15-25oC). The lid of the bottle must be closed between use to ensure maximum potency. Product should be used within 90 days of opening, before the expiry date on bottom of carton.

# Expected Side Effects

The most common side effects of RepHresh Pro-B reported in the literature, which were rare and similar to those reported in placebo groups, were abdominal discomfort, vomiting, or vaginal discomfort.

# Study Products, Labeling, Supply and Accountability

Bay Area Research Logistics (BARL) has been contracted to purchase, package into identical looking kits, label for investigational use, and distribute the study product to the study site.

BARL will take Estring and/or 60 capsules of RepHresh Pro-B based on the four treatment arms below, label them as investigational products in accordance with applicable regulatory requirements, put them in identical cartons, seal the cartons, and label the cartons with a unique sequential kit number. .

- - - - - Group 1: Estring alone
        - Group 2: Estring + RepHresh Pro-B capsules (administered vaginally)
        - Group 3: Estring + RepHresh Pro-B capsules (administered orally)
        - Group 4: RepHresh Pro-B capsules (administered vaginally)

BARL will perform all packaging and logistical operations in accordance with Good Manufacturing Practices standards. An appropriate level of quality assurance and quality control checks will be determined and implemented. Once the study kits have been prepared, BARL will ship the order to the study site (WHIWH clinic). All products will be kept in a locked cupboard and all dispensed study kits will be recorded in a drug- dispensing log. Study kits will be dispensed at the baseline visit. Any unused kits, or parts- thereof will be returned to WHIWH, and BARL will arrange the collection and destruction of study products following the appropriate guidelines.

# Regimen, Administration and Duration

Study participants will be randomized to one of the four treatment groups described below:

1. Group 1: Estring Alone.

Participants will be given a single packet containing one Estring vaginal ring. Participants will begin using the vaginal ring on day 0 and keep it in place until day

30. Estring should not be removed during the one-month intervention period.

1. Group 2: Estring + Twice Daily Vaginal RepHresh Pro-B.

Participants will be given a single packet containing one Estring vaginal ring and a 30 day supply of RepHresh Pro-B probiotic pills. Participants will begin using the vaginal ring on day 0 and keep it in place until day 30. Participants will also be instructed to insert one RepHresh Pro-B capsule vaginally twice daily, morning and night or approximately 12 hours apart until day 30.

1. Group 3: Estring + Twice Daily Oral RepHresh Pro-B.

Participants will be given a single packet containing one Estring vaginal ring, and a 30 day supply of RepHresh Pro-B probiotic pills. Participants will begin using the vaginal ring on day 0 and keep it in place until day 30. Participants will also be

instructed to take one RepHresh Pro-B capsule orally twice daily, morning and night or approximately 12 hours apart until day 30.

1. Group 4: Daily Vaginal RepHresh Pro-B

Participants will be given a 30 days supply of RepHresh Pro-B. On day 0 participants will be instructed to insert one RepHresh Pro-B capsule vaginally twice daily, morning and night or approximately 12 hours apart until day 30.

# Concomitant Medications/Natural Remedies/Foods

Concomitant medications and reported natural remedies and supplements should be recorded in the Case Report Forms (CRFs).

No formal Drug-Drug Interaction studies with Estring have been conducted. The following drug-interactions are based on the experience of systemic estrogen treatment; Estring is a local vaginal estrogen therapy. Participants will be informed of the following interactions but cautioned that these have not been studied with the use of Estring and no restrictions, other than previously discussed exclusion criteria are in place for this study.

- - - - - Estrogen may diminish the effectiveness of anticoagulant, antidiabetic and antihypertensive agents.
        - Preparations inducing liver enzymes (e.g. barbiturates, hydantoins, carbamazepine, meprobamates, or rifampicin) can enhance estrogen metabolism, resulting in breakthrough bleeding or vaginal spotting.
        - In vitro and in vivo studies have shown that systemic estrogens are metabolized partially by cytochrome P450 3A4 (CYP3A4). Therefore, inducers or inhibitors of CYP3A4 may affect estrogen metabolism.
        - Inducers of CYP3A4 such as phenobarbital, carbamazepine, and rifampin may reduce plasma concentrations of estrogens, possibly resulting in a decrease in systemic effects and/or changes in the uterine bleeding profile.
        - Inhibitors of CYP3A4 such as erythromycin, clarithromycin, ketoconazole, itraconazole, ritonavir may increase plasma concentrations of estrogens and may result in side effects. Ritonavir and nelfinavir, although known as strong inhibitors, by contrast exhibit inducing properties when used concomitantly with steroid hormones.
        - Use of ESTRING should be discontinued during treatment with vaginal antimicrobial therapy.
        - Inhibitors of CYP3A4, such as grapefruit juice, may increase plasma concentrations of estrogens and may result in side effects.
        - Inducers of CYP3A4 such as St. John’s Wort preparations (Hypericum perforatum) may reduce plasma concentrations of estrogens, possibly resulting in a decrease in systemic effects and/or changes in the uterine bleeding profile.
        - It was found that some herbal products (e.g. St John’s Wort) which are available as over-the-counter products might interfere with steroid metabolism and therefore alter the efficacy and safety of estrogen/progestin products.

RepHresh Pro-B probiotic pills contains live cultures of Lactobacillus rhamnosus (GR-1) and Lactobacillus reuteri (RC-14). Taking antibiotics along with the probiotic will reduce the effectiveness of these strains. Participants should avoid taking antibiotics as they may reduce the activity of RepHresh Pro-B however, if a participant is prescribed antibiotics, study staff must be notified and the use of antibiotics should be recorded in the CRFs. There are no other restrictions in place for other medications, natural remedies or foods for participants taking RepHresh Pro-B.

# Concomitant Alcohol and “Street” Drug Use

No restrictions are placed on alcohol or illicit drug use and will not be monitored throughout the study.

# Prohibited Medications and Procedures

The use of oral antibiotics and oral steroids is prohibited within 30 days of the Baseline Visit (Visit 2), unless needed to treat any intercurrent medical condition. Any hormonal medications, including hormonal birth control, are prohibited 3 months prior to and during the course of the study; IUD use is not permitted. Individuals taking immunosuppressive drugs such as (Heazathioprine (Imuran), basiliximab (Simulect), cyclosporine (Neoral, Sandimmune), daclizumalthab (Zenapax), muromonab-CD3 (OKT3, Orthoclone OKT3), mycophenolate (CellCept), tacrolimus (FK506, Prograf), sirolimus (Rapamune), prednisone (Deltasone, Orasone), or oral corticosteroids (glucocorticoids) are excluded from the study (see exclusion criteria).

Concomitant medication which in the opinion of the investigator may be associated with a significant drug interaction with the estrogen in Estring are prohibited.

# Participant access to study medication at study closure

The study treatments will not be provided after study closure. Treatments are experimental and currently there is no known benefit to providing these treatments after study completion. RepHresh is available for purchase “over-the counter”; Estring is a prescription drug and its use in pre-menopausal women is considered “off-label”.

# RISKS AND PRECAUTIONS

# Acceptable Methods of Birth Control

While abstinence from sexual activity is the only certain method to prevent pregnancy, female participants of childbearing potential who are or who anticipate the possibility of becoming sexually active with a male partner during the study must practice an acceptable method of contraception, defined for the purpose of this study as a barrier method (acceptable barrier methods include diaphragm, or male or female condom), unless their male partner has had a previous vasectomy.

Contraceptive measures will be reviewed with participants at all study visits over the course of the study.

# Mental Health Support

The study site, Women’s Health in Women’s Hands (WHIWH) is a community health clinic with mental health support services and personnel available within the clinic. Should there be any need, study participants can be referred to mental health services within the clinic during participation in the study or upon completion.

# Risk Management

Risk minimization, management, and assessment procedures have been implemented in the study to minimize and assess potential risks to participants who participate in this clinical study with Estring and RepHresh Pro-B Probiotic. Components include: (1) specific study entry and exclusion criteria to ensure that participants who have underlying characteristics that potentially increase their risk for an adverse outcome are excluded;

(2) protocol-specific procedures for minimizing and managing certain AEs, e.g. one month treatment intervention, qualified personnel performing all study evaluations and procedures; (3) overview surveillance by an independent Data Safety Monitoring Committee; (4) ongoing follow-up for 7 days for safety monitoring purposes.

# CLINICAL AND LABORATORY EVALUATIONS

# Clinical Evaluations

At screening (Visit 1) a routine medical assessment (including medical history and current medication) will be conducted. The physical examination will include vital signs, head and neck, lung, cardiovascular and abdominal exam. At each study visit a directed physical examination will be conducted. In addition, participants will be asked about any adverse effects and any changes in their health, or newly diagnosed medical conditions or new medications started since their last study visit.

# Pelvic Examinations

Pelvic exams will be performed at screening and all study visits. The study nurse/doctor at the study site will lubricate a vaginal speculum with normal saline and insert into the vaginal canal gently. The external and internal genitalia and surrounding tissues will be examined for any signs of infection (redness, inflammation, discharge, foul odour, lesions, etc.).

# Laboratory Evaluations and Specimen Collection

# Clinical Laboratory Tests

At screening (Visit 1) a urine sample for gonorrhea and chlamydia testing, and a blood sample for HIV testing will be collected, processed, stored and shipped to the local testing laboratory as per standard-of-care. The urine pregnancy test will be performed on site by the research staff. Participants will be provided with the results of the clinical laboratory tests. Safety bloods (glucose, Ca, non-fasting triglycerides and cholesterol, alanine aminotransferase (ALT), bilirubin, alkaline phosphatase (ALP), Na, K, creatinine, CBC) will be collected and sent to the local testing laboratory as per standard of care. Participants with any clinically significant abnormalities at screening that in the opinion of the investigator would preclude their enrolment, will be excluded from the study and referred to their physician for medical follow-up as necessary. Safety bloods will be repeated at the end of treatment (Visit 4) and any clinically significant abnormality referred for follow-up as necessary.

Table 3: Clinical Laboratory Tests:

| **Test** | **Method** | **Sample** | **Laboratory** |
| --- | --- | --- | --- |
| Pregnancy test | hCG detection | Urine | Point-of-care WHIWH |
| Neisseria gonorrhoea | NAAT | Urine | TPHL |
| Chlamydia trachomatis | NAAT | Urine | TPHL |
| HIV antibody | EIA | Blood | TPHL |
| Safety bloods | Routine analyses | Blood | Gamma-Dynacare Lab |

# Gonorrhea/Chlamydia Testing

First catch urine will be collected at the research site in a sterile urine collection cup. Subsequently 2mL of urine will be transferred from the collection cup to the Aptima Urine Specimen Transport Tube (Hologic, Toronto, ON) using the pipette provided with the Tube. Urine will be refrigerated and sent to Public Health Ontario for NAAT testing.

# HIV Serology

Blood will be collected by the study nurse from the cubital vein by venipuncture. Blood will be sent to Public Health Ontario for HIV testing.

# Urine Pregnancy Test

Estring treatment is contraindicated with pregnancy, and all women will have a pregnancy test at Visit 1 (screening) and again at Visit 2 (baseline) prior to randomization.

Urine will be collected at the research site in a sterile urine collection cup. Using the pipette provided in the pregnancy testing kit, 3 drops of urine will be placed on the test area, and left to sit for 3 minutes before reading. Testing will occur at the research site and the result will be interpreted by the study nurse.

# Research Sample Collection and Processing

All necessary lab materials e.g. blood tubes, cytobrush, slides, labels, shippers, etc. will be supplied to the research site.

Samples will be collected at study Visit 2, 4 and 5 and shipped by courier to McMaster Immunology Research Centre for further testing and analysis as follows:

# Blood Collection

Blood (20-40 mL) will be collected at Baseline (Visit 2) and End of Treatment visit (Visit

4) by the study nurse. Samples will be refrigerated at 2-8°C until samples are packed for shipment. Blood will be sent to McMaster University for chemokine/cytokine/immune protein/hormone detection and at baseline for HSV antibody screening.

# Immunology Phenotyping and HSV serology

Peripheral blood mononuclear cells (PBMCs) will be extracted by Ficoll-Paque density gradient centrifugation for immunology phenotyping by flow cytometry and cell culture assays, and frozen at -80°C for batch analysis.

Whole blood collected in plasma separator tubes during blood collection will be centrifuged to isolate plasma. Plasma will be aliquoted and stored for batch analysis of chemokine, cytokines, immune protein and hormone detection as well as baseline HSV I and 2 antibody using HerpesSelect HSV-1/2 Immunoblot assay (Focus Diagnostics, CA, USA).

# Self-collected Vaginal Swab

At Visit 2 (baseline), participants will be given an instruction sheet, a Copan Diagnostic Flocked Swab, and gloves by the study nurse at the study site. They will be instructed to don gloves, insert swab into vagina at least 1 inch, and rotate the swab 360 degrees, privately during this visit. The swab will be placed back in its tube and the gloves will be disposed. Participants will be sent home with the aforementioned items to collect additional swabs between 7 days after the clinic visit and 24 hours prior to visits 3 and 4. Swabs will be brought to the study nurse and shipped on ice to McMaster University for testing. The self-collected swab specimens will be collected at visits 2, 3, and 4. Self- collected vaginal swabs will be used for microbiome analysis.

Study participants are required to abstain from vaginal intercourse for 48 hours prior to vaginal swab collection, over the entire course of the study to avoid the potential effect of sexual activity/semen on the analysis of samples for testing the vaginal microbiome and immune/inflammatory markers.

# Vaginal/cervical samples collected during pelvic examination PSA testing

Prior to cervical sampling at Visits 2 (baseline), 3, 4 and 5 a vaginal swab will be

collected for PSA testing and shipped along with the other samples to the McMaster for batch testing. A rapid prostate-specific antigen testing kit (Seratec PSA

Semiquant, Gottingen, Germany) will be used to determine recent exposure to PSA (recent unprotected intercourse).

Each swab will be placed in 500µl extraction buffer from the testing kit and vortexed for 30 seconds. Subsequently approximately 100µl of the solution will be loaded directly onto the kit test strip using the pipette provided in the kit. Results will be read approximately 10 minutes later and recorded in study documentation. Results will not be provided to participants.

# Vaginal Swabs (BV, Microbiome/HSV, PSA)

During the pelvic exam the study nurse will gently insert and rotate Copan Diagnostic Flocked Swabs (3 swabs at baseline, and 2 swabs at visits 3, 4, and 5) in all four quadrants of the posterior vaginal fornix, as well as the cervical os. The swab for Microbiome/HSV-2 and PSA testing will be placed back in its tube, refrigerated, and shipped to McMaster University on ice. The swab for BV testing (baseline visit only) will be smeared on a glass slide, fixed in methanol, air-dried, placed in a slide mailer, and shipped to McMaster University. The slide will be stained and the Nugent Score calculated.

# Cervical Sampling (Cytobrush, CVL)

Cytobrush and CVL samples will be collected at Visits 2 (baseline), 3, 4, and 5. During the pelvic exam the study nurse will gently insert a cytobrush into the cervical os, rotate 360 degrees, and remove. The cytobrush will be placed in a 15mL Falcon tube containing 5mL of sterile PBS, and refrigerated. The cervical os will also be washed with 2mL of sterile saline using a sterile syringe. The liquid will be aspirated from the vaginal canal back into the syringe and the process of washing the cervical os will be repeated 4 more times. The CVL sample will be placed in a sterile 2mL vial, and spun by centrifuge for 30s. The supernatant will be collected and divided into four 0.5mL aliquots, each placed in a 2mL sterile tube. Cytobrush and CVL samples will be shipped on ice to McMaster University for characterization of cervical immune cell populations, innate soluble factor detection, and microbiome analysis.

# Characterization of Cervical Immune Cell Populations from Cytobrush

Once Cytobrushes arrive at McMaster University they will be frozen at -80°C for batch isolation of cervical mononuclear cells (CMC). These specimens will be used to characterize the cervical immune cell populations by flow cytometry.

# Innate Soluble Factor Detection and Microbiome Analysis from CVLs

After CVLs arrive at McMaster, they will be frozen at -80 for batch processing. CVLs will be used for innate soluble factor detection (chemokines, cytokines, antibodies, innate protein, etc.) using methods like ELISA. They will also be used for microbiome analysis (PCR amplification of 16S rRNA gene, NextGen barcoded sequencing, data analysis) through the McMaster Genomics Facility.

Study participants are required to abstain from vaginal intercourse for 48 hours prior to sample collection, over the entire course of the study to avoid the potential effect of sexual activity/semen on the analysis of samples for testing the vaginal microbiome and immune/inflammatory markers.

# Research Laboratory Assays

# Microbiome analysis from CVL samples:

Aliquots of CVL sample will be stored at -70oC for molecular analysis of the microbiota. DNA extraction will be carried out using a previously described protocol that enhances DNA recovery from microbial communities ([Sibley et al., 2011](#_bookmark169); [Sibley et al., 2008](#_bookmark170)) with modifications ([Whelan et al., 2014](#_bookmark175)) to increase quantitative recovery of bacteria across different taxa. Bacterial community profiling of 16S rRNA gene will be carried out using a modified bar coded Illumina sequencing method of Bartram et al., ([Bartram et al., 2011](#_bookmark118)) generating overlapping paired end reads of the V3-V4 region using the 341F ([Muyzer et](#_bookmark158) [al., 1993](#_bookmark158)) and 806R ([Relman et al., 1990](#_bookmark165)) primers (using 300nt paired-end sequencing on an Illumina MiSeq). Using the full 96 barcodes will provide approximately 50-100,000 16s rRNA reads per sample. The MiSeq data will be processed by an in-house bioinformatics pipeline ([Whelan et al., 2014](#_bookmark175)) that incorporates quality filtering, cutadapt ([Martin, 2011](#_bookmark149)), PandaSeq ([Masella et al., 2012](#_bookmark152)), Abundant OTU ([Ye, 2011](#_bookmark177)), mother ([Schloss et al., 2009](#_bookmark166)), and QIIME ([Caporaso et al., 2010](#_bookmark123); [Lozupone et al., 2011](#_bookmark147)). Taxonomic assignments will use the RDP classifier ([Wang et al., 2007](#_bookmark173)) using the Greengenes training set ([DeSantis et al., 2006](#_bookmark125); [McDonald et al., 2012](#_bookmark154)). Microbiome analysis will include α-diversity metrics for each sample and β-diversity measures (weighted and unweighted unifrac, Bray-Curtis, nonmetric multidimensional scaling) and other statistical analysis using QIIME ([Caporaso et al., 2010](#_bookmark123); [Lozupone et al., 2011](#_bookmark147)), PhyloSeq ([McMurdie and Holmes, 2013](#_bookmark155)) and LEfSe([Aagaard et al., 2012](#_bookmark116)). A microarray technique developed in Gregor Reid’s lab will be used for assessing several species of lactobacilli, as previously described, and we will add L. gasseri, L. rhamnosus and L. reuteri to the 8 species template ([Dols et al., 2012](#_bookmark126)). In addition, we will use qPCR to determine abundance of GR-1 and RC-14 strains.

# Innate inflammatory cytokine/chemokine analysis

The impact of local estrogen on the inflammatory environment of the female genital tract will be assessed by measuring the level of cytokine/chemokines in CVL by multiplex assay (Milliplex MAP Human Cytokine Kit I, II kits) and measured by Luminex technology. This instrument and technology are available at McMaster University. We typically perform a 22-plex that measures a range of cytokines and chemokines. Statistical tests will be done to assess the normal distribution of the data and a non-parametric one-way analysis of variance will be done if variables are not normally distributed between groups. The panel of analytes we will use has been used in previous studies to demonstrate an increased state of mucosal activation and inflammation in different groups of women from the Pumwani cohort ([Lajoie et al., 2012](#_bookmark146)).

# HIV Target cell assay

We recently constructed a β-lactamase-Vpu (BLaM-Vpu)-containing HIV pseudovirus to quantify HIV susceptible CD4+ T cells in the female genital tract, which is pseudotyped with an early transmitted, CCR5-tropic, Clade A or B envelope([Joag et al., 2016](#_bookmark138)) with target cells delivers BlaM-Vpr into the cytosol, and cells are then loaded with the substrate CCF2-AM, a membrane-permeant form of the fluorescent molecule CCF that contains two fluorophores, 7-hydroxy-coumarin and fluorescein, linked by a β-lactam bond. In the absence of β-lactamase, excitation of 7-hydroxycoumarin at 409nm leads to fluorescent resonance energy transfer (FRET) to fluorescein and green light emission at 520nm. In infected cells, β-lactamase (delivered by BlaM-Vpr) cleaves the β-lactam bond of CCF2- AM, preventing FRET, and causing blue emission (447nm) by 7-hydroxy-coumarin. Therefore, the ratio of blue to green emission is a measure of viral fusion and cytosolic entry of HIV. This assay can rapidly and reproducibly quantify cervical CD4+ T cell number and/or subsets infected by HIV in cytobrush samples, without *in vitro* cell activation. The cytobrush samples will be sent to University of Toronto lab for HIV target testing.

# Immunology phenotyping, etc on peripheral blood

Peripheral blood mononuclear cells will be isolated using the standard Ficoll procedure. Isolated PBMCs will be frozen in -80oC for batch processing. Immune cells isolated from blood will be tested for innate antiviral response in cell culture assays and stained with a panel of antibodies against T cells and other immune cells phenotyped by flow cytometry analysis. An aliquot of the blood will be used for separating serum for HSV 1/2 analysis. The analysis will be done in batches using a commercial using Herpes Select HSV-1/2 Immunoblot assay (Focus Diagnostics, CA, USA).

# Stored Research Specimens

Only the study team will have access to participants’ study samples. The samples will be analyzed and stored at McMaster University in Hamilton, Ontario. Samples collected and stored for this study will be used only for the purpose of this study’s aims. Samples will be stored for the for a maximum duration of 20 years after end of study at McMaster University and will be subsequently destroyed.

# Questionnaires

Participants will complete a Sexual History Questionnaire at enrollment (Study visit 2) and a daily diary for the duration of the study, with help from the study staff as needed.

# STUDY PROCEDURES

- - - 1. **Schedule of Events** **Table 4: Schedule of Events**

| **Visit Number** | **Visit 1** | **Visit 2** | **Visit 3** | **Visit 4** | **Visit 5** |
| --- | --- | --- | --- | --- | --- |
| **Visit** | **Screening** | **Baseline1** | **On Treatment Visit** | **End of Treatment Visit** | **Safety Follow up Visit2** |
| **Visit Day** | - | Day 0 | 14 | 31 | 37 |
| **Visit Window** | Day -45 to -7 | - | ± 3 days | + 3 days | + 3 days |
| Informed Consent | X |  |  |  |  |
| Assessment of Eligibility | X | X |  |  |  |
| Randomization/Enrollment |  | X |  |  |  |
| Physical Exam3 | X | X | X | X | X |
| Medical History | X |  |  |  |  |
| Sexual History Questionnaire |  | X |  |  |  |
| Concomitant Medications |  | X | X | X | X |
| Urine Pregnancy test | X | X |  |  |  |
| Gonorrhea/Chlamydia testing4 | X |  |  |  |  |
| Diary Distribution |  | X |  |  |  |
| Diary Review |  |  | X | X | X |
| Diary Return |  |  |  | X | X |
| Self-collected vaginal swab specimen collection5 |  | X | X | X |  |
| Study products dispensation |  | X |  |  |  |
| Return of study products |  |  |  | X |  |
| Assessment of AEs |  | X | X | X | X |
| Assessment of compliance |  |  | X | X |  |
| Safety bloods | X |  |  | X |  |
| HIV serology | X |  |  |  |  |
| Blood collection6 |  | X |  | X |  |
| Pelvic exam | X | X | X | X | X |
| PSA test7 (cervical/vaginal swab) |  | X | X | X | X |

1 Scheduled 5-10 days after 1st day of menstrual cycle

2 See protocol for procedure to be completed if participant has consented to attending an “in-clinic” visit.

3 A directed physical examination will be performed at subsequent visits after the screening visit

4 Urine NAAT

5 Self-collected vaginal swab will be done during visit 2, and a maximum of 24 hrs prior to visits 3 and 4 6 20 - 40 mL blood (four tubes) collected for PBMCs for immunology phenotyping, DNA isolated for genetic analysis, plasma for chemokine/cytokine/immune protein/hormone detection, HSV screening at baseline only

7 Prostate-specific antigen (PSA) test to determine unprotected sex in past 48 hours. Swab collected prior to collection of cervical samples.

| **Visit Number** | **Visit 1** | **Visit 2** | **Visit 3** | **Visit 4** | **Visit 5** |
| --- | --- | --- | --- | --- | --- |
| **Visit** | **Screening** | **Baseline1** | **On Treatment Visit** | **End of Treatment Visit** | **Safety Follow up Visit2** |
| **Visit Day** | - | Day 0 | 14 | 31 | 37 |
| **Visit Window** | Day -45 to -7 | - | ± 3 days | + 3 days | + 3 days |
| Vaginal swab (BV) |  | X |  |  |  |
| Vaginal swab (microbiome/HSV) |  | X | X | X | X |
| Cervical sampling (CMC, CVL) |  | X | X | X | X |

# Study Visits

- - 1. **Visit 1: Screening Visit (Day -45 to -7)**

The screening visit is anticipated to take approximately 1 hour. During this visit, the following with take place:

- Informed consent
- Review of eligibility criteria
- Collection of demographic information and medical history
- Physical examination
- Urine collected for:
  - Gonorrhea/chlamydia testing and pregnancy test
- Blood (~20 mL) collected for HIV serology and safety monitoring (glucose, Ca, triglycerides, cholesterol, ALT, bilirubin, alkaline phosphatase, Na, K, creatinine, CBC)
- Pelvic exam - external and internal visual exam for signs of redness, irritation, vaginal discharge, cysts, genital warts, ulceration or other clinical abnormalities

# Visit 2: Baseline Visit (Day 0)

A baseline visit will be scheduled if the participant meets all eligibility criteria.

This visit must occur within 45 days of the screening visit and scheduled 5-10 days after the first day of the participant’s menstrual cycle following the screening visit.

This visit is anticipated to take approximately 2 hours. During this visit, the following with take place:

- Confirmation of informed consent
- Confirmation that eligibility criteria has been met
- Directed physical examination
- Concomitant medications
- Completion of Participant questionnaire:
  - Sexual History questionnaire (Section 16.1)
- Study diary distribution
- Urine collected for pregnancy test
- Self-collected vaginal swab
- Blood collection (20-40 mL) for immunology assays and HSV serology
- Pelvic Exam - external and internal visual exam for signs of redness, irritation, vaginal discharge, cysts, genital warts, ulceration or other clinical abnormalities
- Vaginal swab for PSA testing, prior to other cervical/vaginal sampling
- Vaginal swab for BV
- Collection of cervical/vaginal samples for :
- Cervical Mononuclear cells (CMC) for characterization of the cervical immune cell population
- Cervico-vaginal lavage (CVL) for innate soluble factor detection and microbiome analysis
- Copan Diagnostic Flocked swab of lateral vaginal wall for microbiome analysis and/or HSV-2 testing
- Randomization
- Study product dispensation
- AE assessment
- Provide swab for self-collection and instructions for collection of vaginal sample in 5-10 days after clinic visit 2

# Visit 3 (Day 14 ± 3 days) – On Treatment

Visit 3 is anticipated to take approximately 1 hour. During this visit, the following with take place:

- Concomitant medications
- Diary review
- Directed physical examination
- Self-collected vaginal swab (1) return (collected at home between visits 2 and 3)

o Collected between 7 days after the clinic visit and 24 hrs prior to Visit 3

- Provide swab for self-collection and instructions for collection of vaginal sample in 5-10 days after clinic visit 3
- AE assessment
- Assessment of compliance (verbal report).
- Pelvic Exam - external and internal visual exam for signs of redness, irritation, vaginal discharge, cysts, genital warts, ulceration or other clinical abnormalities
- Cervical/vaginal swab for PSA testing, prior to other cervical/vaginal sampling
- Collection of cervical/vaginal samples:
- Cervical Mononuclear cells (CMC) for characterization of the cervical immune cell population
- Cervico-vaginal lavage (CVL) for innate soluble factor detection and microbiome analysis
- Copan Diagnostic Flocked swab of lateral vaginal wall for microbiome analysis and/or HSV-2 testing

# Visit 4 (Day 31 +3 days) – End of Treatment

This visit is anticipated to take approximately 1 hour. During this visit, the following with take place:

- Concomitant medications
- Diary review and collection
- Directed physical examination
- Self-collected vaginal swab (1) return (collected at home between visits 3 and 4)

o Collected between 7 days after the clinic visit and 24 hrs prior to Visit 4

- Return of used Estring as applicable, all unused study products, and study product containers
- AE assessment
- Assessment of compliance (verbal report/diary review/returned medication count)
- Collection of safety bloods (~20mL)
- Blood collection (20-40 mL) for immunology.
- Pelvic Exam - external and internal visual exam for signs of redness, irritation, vaginal discharge, cysts, genital warts, ulceration or other clinical abnormalities
- Cervical/vaginal swab for PSA testing, must be done prior to other cervical/vaginal sampling
- Collection of cervical/vaginal samples:
- Cervical Mononuclear cells (CMC) for characterization of the cervical immune cell population
- Cervico-vaginal lavage (CVL) for innate soluble factor detection and microbiome analysis
- Copan Diagnostic Flocked swab of lateral vaginal wall for microbiome analysis and/or HSV-2 testing

# Visit 5 (Day 37 ± 3 days) – Safety Follow-up

This visit is will take place by phone, or at an in-clinic visit if the participant consents to additional sample collection. It is anticipated this visit to take approximately 30 minutes (by phone) or 1 hour (in-clinic visit).

During this visit, the following with take place:

- Concomitant medications
- AE assessment

# In-clinic visit (optional)

- Diary review and return
- Directed physical examination
- Pelvic Exam - external and internal visual exam for signs of redness, irritation, vaginal discharge, cysts, genital warts, ulceration or other clinical abnormalities
- Cervical/vaginal swab for PSA testing, must be done prior to other cervical/vaginal sampling
- Collection of cervical/vaginal samples:
- Cervical Mononuclear cells (CMC) for characterization of the cervical immune cell population
- Cervico-vaginal lavage (CVL) for innate soluble factor detection and microbiome analysis
- Copan Diagnostic Flocked swab of lateral vaginal wall for microbiome analysis and/or HSV-2 testing

# Early Termination Visit

If a participant misses her study visit(s), a study team member from the clinic will try to contact her. Even participants who stop taking the study treatment for any reason will be asked to complete the follow-up visits through to Visit 4. If a participant terminates the study early, the following procedures should be conducted:

- Procedures and evaluations scheduled for the Final Study Visit (Visit 4) are performed, including assessments of AEs.
- Study treatment compliance is assessed
- Any study products are returned and inventoried The reason for study termination will be recorded.

For participants who remain in the study without taking the study drug, in addition to the procedures outlined in Table 4, the study coordinator or investigator will ask questions to determine how the participant has been doing since stopping the study, as a safety measure. If a participant develops side effects from study drug, the investigator may want to see her more often than the scheduled visits. Additional blood samples for safety monitoring purposes, based on the investigator’s opinion, may be needed.

# EVALUATION, RECORDING, AND REPORTING OF ADVERSE EVENTS

# Definitions

# Adverse Event (AE)

An AE is any untoward medical occurrence in a patient or clinical investigation participant, administered a study medication/intervention, which does not necessarily have a causal relationship with this treatment. An AE can therefore be any unfavorable and unintended sign (including an abnormal laboratory finding), symptom, or disease temporally associated with the use of a medicinal (investigational) study medication/intervention, whether or not related to the medicinal (investigational) study medication/intervention.

Some of the AEs that could be expected are as follows:

- - - - Increased vaginal secretions
      - Vaginal discomfort
      - Abdominal pain
      - Urogenital itching
      - Vaginal bleeding/spotting
      - Headache
      - Breast tenderness
      - Fluid retention/leg edema
      - Increased gas or bloating
      - Nausea or vomiting
      - Diarrhea
      - Heartburn

During each follow-up visit with the participant, information on AEs will be gathered and documented accordingly. AEs will be graded as mild, moderate, severe or life threatening and assessed by causality as probably related, possibly related, unlikely to be related or not related to the study intervention.

Stable chronic conditions which are present prior to clinical trial entry and do not worsen are not considered AEs and will be accounted for in the participant’s medical history.

# Serious Adverse Events (SAEs)

An SAE is defined as an AE meeting one of the following criteria at any dose:

- - - - Results in death during the period of protocol-defined surveillance
      - Is a life-threatening event (defined as a participant at immediate risk of death at the time of the event)
      - Results in in-patient hospitalization or prolongation of existing hospitalization during the period of protocol-defined surveillance
      - Results in persistent or significant disability or incapacity. (Disability is defined as a substantial disruption of a person's ability to conduct normal life functions)
      - Is a congenital anomaly or birth defect

Any other important medical event that may not result in one of the above outcomes, may be considered a SAE when, based upon appropriate medical judgment, the event may jeopardize the participant and may require medical or surgical intervention to prevent one of the outcomes listed above. Examples of such medical events include allergic bronchospasm requiring intensive treatment in an emergency room or at home, blood dyscrasias or convulsions that do not result in in-patient hospitalization, or the development of drug dependency or drug abuse.

Participants will be monitored for SAE’s between the time that a study participant is enrolled and the time that she departs the study at the end of the final study visit If an SAE is ongoing at the time a participant discontinues/completes the study, the SAE will be followed until the Investigator agrees that the event is satisfactorily resolved, becomes chronic, or that no further follow-up is required.

# AE Descriptions and Recording

All Adverse Events reported in the study will be collected in the eCRF, regardless of intensity (severity) or relationship to study treatment.

# Intensity

The Intensity (severity) for each AE (including SAE) will be graded according to the toxicity table and toxicity guidelines provided by the Division of AIDS (DAIDS) Table for Grading the Severity of Adult and Pediatric Adverse Events V2.1 ([https://rsc.tech-](https://rsc.tech-res.com/clinical-research-sites/safety-reporting/daids-grading-tables) [res.com/clinical-research-sites/safety-reporting/daids-grading-tables](https://rsc.tech-res.com/clinical-research-sites/safety-reporting/daids-grading-tables) ).

# Relationship to Study Treatment

For all collected AEs (including SAEs), the clinician who examines and evaluates the participant will determine the AE’s causality based on temporal relationship and his/her clinical judgment. The degree of certainty about causality will be graded using the categories below:

**Definitely Related:** There is clear evidence to suggest a causal relationship, and other possible contributing factors can be ruled out. The clinical event, including an abnormal laboratory test result, occurs in a plausible time relationship to drug administration and cannot be explained by concurrent disease or other drugs or chemicals. The response to withdrawal of the drug (de-challenge) should be clinically plausible. The event must be pharmacologically or phenomenologically definitive, with use of a satisfactory re- challenge procedure if necessary.

**Probably Related:** There is evidence to suggest a causal relationship, and the influence of other factors is unlikely. The clinical event, including an abnormal laboratory test result, occurs within a reasonable time sequence to administration of the drug, is unlikely to be attributed to concurrent disease or other drugs or chemicals, and follows a clinically reasonable response on withdrawal (de-challenge). Re-challenge information is not required to fulfill this definition.

**Possibly Related:** There is some evidence to suggest a causal relationship (e.g., the event occurred within a reasonable time after administration of the trial medication).

However, the influence of other factors may have contributed to the event (e.g., the participant’s clinical condition, other concomitant events). Although an adverse drug event may rate only as “possibly related” soon after discovery, it can be flagged as requiring more information and later be upgraded to “probably related” or “definitely related”, as appropriate.

**Unlikely:** A clinical event, including an abnormal laboratory test result, whose temporal relationship to drug administration makes a causal relationship improbable (e.g., the event did not occur within a reasonable time after administration of the trial medication) and in which other drugs or chemicals or underlying disease provides plausible explanations (e.g., the participant’s clinical condition, other concomitant treatments).

**Not related:** The AE is completely independent of study drug administration, and/or evidence exists that the event is definitely related to another etiology. There must be an alternative, definitive etiology documented by the clinician.

# Reporting and Evaluation of SAEs and Other Clinically Significant AEs

# SAEs

All SAEs which occur during the course of the study must be reported to the CTN within 24 hours of the site becoming aware of the event. CTN will be responsible for reporting expedited, reportable SAEs to Health Canada on behalf of the Sponsor-Investigator.

SAEs will be reported to:

| **CIHR Canadian HIV Trials Network** | |
| --- | --- |
| Attention: | Judy Needham |
| Phone: | 604-682-2344 ext. 63366 |
| Fax: | 604-806-8005 |
| E-mail: | [jneedham@cheos.ubc.ca](mailto:jneedham@cheos.ubc.ca) |

All additional follow-up evaluations must also be reported as soon as possible in the same manner. All SAEs will be followed until the Investigator agrees that the event is satisfactorily resolved, becomes chronic, or no further follow-up is required.

The Qualified Investigator is responsible for ensuring that their local Research Ethics Boards (REB) are notified of any SAEs as per the local requirements.

# Other Clinically Significant AEs (if applicable)

Treatment should be discontinued and a healthcare practitioner immediately contacted in the following situations, and/or if a contra-indication is discovered:

- - - - Jaundice or clinical deterioration in liver function
      - Significant increase in blood pressure
      - New onset of migraine-type headache
      - Pregnancy
      - Digestive upset (e.g. diarrhoea) occurs, worsens or persists beyond 3 days.

# Follow-up for Adverse Events

Any AE that occurs between the time that a study participant is enrolled and the time that she departs the study at the end of the final study visit (or at the time of early discontinuation of the participant from the study for any reason) will be captured and recorded. At each contact with the participant, the investigator (or designate) must seek information on AEs by specific questioning and, as appropriate, by examination*.*

AEs that had previously been reported by the study participant will also be reassessed for duration, intensity and possible reoccurrence. Assessment of safety will include clinical observation and questions about changes in medication and any new symptoms reported. Specific questions will be asked about any new vaginal symptoms and GI symptoms.

All AEs (including SAEs) will be followed until resolution or until the investigator and the clinical/medical monitor are in agreement that the AE has resolved, stabilized or become chronic and no further follow-up is required.

# Pregnancy Reporting

If a participant becomes pregnant during the study, the participant will be instructed to stop treatment as soon as they are aware of the pregnancy and inform the investigator.

If pregnancy occurs during the period of protocol-defined surveillance, it is not to be considered an AE or SAE requiring monitoring and follow up; however, any complication related to pregnancy would be considered an adverse event. Pregnancy should be recorded as a protocol deviation.

The investigator must document the pregnancy information, birth and status of the child using the pregnancy CRF. The pregnancy must be followed to determine the outcome (including premature termination) and the status of the mother and of the child. Pregnancy complications and elective terminations for medical reasons must be reported as an AE or as an SAE. Spontaneous abortions must be reported as an SAE.

Any SAE that occurred in association with a pregnancy that was brought to the investigator’s attention after the participant completed the study and that was considered by the investigator as being possibly related to the investigational product must be promptly reported. Refer to Section [10.1.2](#_bookmark77).

# Stopping rules

Further enrolment into the study will be suspended if:

1. One or more participants have experienced a SAE probably or definitely related to the study intervention, or
2. In one or more participants, occurrence of Grade 3 or greater AEs judged probably or definitely related to the study intervention.

Within 14 days of suspension of further enrolment because of adverse events, the Data Safety and Monitoring Committee (DSMC) will be convened with the PI’s to review and discuss the safety data. There must be a unanimous decision by all meeting participants to restart enrolment or to permanently discontinue further enrolment. A recommendation will also be made on the management of participants currently in the study.

# STATISTICAL CONSIDERATIONS

# General Study Design

This prospective, randomized, open-label intervention pilot study will enrol a cohort of 80 African, Caribbean and Black (ACB) women, with each woman serving as her own control. Participants will be randomized 1:1:1:1 to receive treatment with 1) a low dose estradiol vaginal ring alone, 2) a low dose estradiol vaginal ring in combination with a vaginal probiotic, 3) a low dose estradiol vaginal ring with an oral probiotic, or 4) a vaginal probiotic alone.

# Sample Size Considerations/Justification

This is a Phase 1 pilot study to establish the feasibility, tolerability and safety of the administration of Estring and RepHresh Pro-B probiotics, the feasibility of enrolling participants, and to gather preliminary immunological and microbiome data to inform the design of a larger trial.

It has been estimated that 40 – 50% of women enrolled will have bacterial vaginosis, providing 8 – 10 women per group in whom the effect of the intervention on local immunological responses and the vaginal microbiome can be evaluated. There has been no formal sample size calculation performed.

Enrolment for the present study is expected to take 12 months.

# Data Sets to be Analyzed

All participants who are enrolled will contribute to the feasibility, demographic and baseline data outcomes. All participants who are randomized to the intervention and receive any treatment, including those who withdraw from the study or deviate from the protocol, will contribute to the data safety set. In the safety analysis, no data exclusion is allowed because of protocol deviations. All participants who received any treatment will contribute to the immunological and microbiome data set; data from participants who complete the protocol without significant intervention will form the main efficacy outcome. All safety and laboratory analyses will be performed without knowledge of the allocation group.

# Endpoints/Outcome Measures

Primary Endpoint:

- - 1. The primary endpoint of this study are the feasibility of enrolling participants and the safety and tolerability of low dose estrogen treatment in combination with probiotics. Feasibility will be evaluated by determining the number of eligible participants approached who consent to be enrolled (screening log), retention rate/dropouts/withdrawal from study, adherence to the protocol, the completion rate (the number of enrolled participants who complete the study protocol, with and without protocol violations), and data completion rate, including completion of diaries and questionnaires. A target of 70% of eligible participants consenting and 80% completing the study is judged acceptable. Safety and tolerability will be evaluated by review of case report forms (CRF), questionnaires, adverse events (AE), serious adverse events (SAE) and treatment adherence. Adherence will be measured by calculating the total probiotic used/total dispensed and for Estring adherence will be measured by total days used/total time period.

Secondary Endpoints:

The secondary endpoints of the study will be to observe changes in *Lactobacillus* species (enhanced proportion of lactobacilli) in the vaginal microbiota following Estring and oral/vaginal RepHresh treatment. Additional endpoints evaluated will include the change in inflammatory factors in the CVL and blood and HIV target T cells in the FGT, following intervention.

# Analysis of Demographic and Baseline Data

Tabulations and descriptive statistics will be reported for demographic and baseline data collected through the screening visit interview and participant questionnaire.

# Analysis of Primary Outcome Measures

Descriptive statistics will be used to report feasibility outcomes.

The screening log will include information on why participants declined to be enrolled.

The Intensity (severity) for each AE (including SAE) will be graded according to the toxicity table and toxicity guidelines provided by the Division of AIDS (DAIDS) Table for Grading the Severity of Adult and Pediatric Adverse Events V2.1 ([https://rsc.tech-](https://rsc.tech-res.com/clinical-research-sites/safety-reporting/daids-grading-tables) [res.com/clinical-research-sites/safety-reporting/daids-grading-tables](https://rsc.tech-res.com/clinical-research-sites/safety-reporting/daids-grading-tables) ).

Tabulations and descriptive statistics will be employed in the analysis of all safety and tolerability observations.

# Analysis of Secondary Outcome Measures

Changes in outcome parameters will be compared prior to and post treatment with each participant acting as their own control. Statistical significance of change in microbiome populations will be done by comparison α-diversity metrics for each sample and β- diversity measures (weighted and unweighted unifrac, Bray-Curtis, nonmetric multidimensional scaling), association analysis (indicator species, co-occurrence/mutual exclusion) and other statistical analyses using QIIME ([Caporaso et al., 2010](#_bookmark123); [Wang et al.,](#_bookmark173) [2007](#_bookmark173)), PhyloSeq ([Aagaard et al., 2012](#_bookmark116)) and LEfSe ([Dols et al., 2012](#_bookmark126)). Significance of changes in levels of pro-inflammatory cytokines in CVL will be done by comparing continuous distributions using rank correlation and comparing cytokines by high (highest quartile) or low expression using the Mann-Whitney-Wilcoxon test. Cytokine/inflammatory factor levels as a continuous measure will be compared by experimental treatment using linear regression, using the final score as the dependent variable, treatment group as the independent variable, and the initial score as a baseline covariate. If the data fail to meet assumptions underlying linear regression even after data transformation, nonparametric ANOVA (Kruskal-Wallis test) will be used. Spearman test will be performed to determine the correlation between immune inflammation in the CVL and microbiome analysis results. Changes in T cells populations will be done by flow analysis and applying t-test.

# STUDY ETHICAL CONSIDERATIONS

# Ethical Conduct of the study

This study will be conducted in accordance with the ICH GCP Guidelines, applicable Health Canada regulations, and the principles of the Declaration of Helsinki. The Sponsor-Investigator will be thoroughly familiar with the appropriate use of the study treatment as described in the protocol.

The study protocol, informed consent forms and addition study-related documentation will be submitted for approval to the Hamilton Integrated Research Ethics Board. A Clinical Trial Application will be submitted to Health Canada’s Therapeutic Products Directorate. All participants will be given adequate explanation of the aims, methods, anticipated benefits and potential risks of the study. Participants will be required to sign an informed consent form prior to the initiation of the screening process.

# Informed Consent

All participants will be given detailed oral and written information about the study. Consent forms describing in detail the study medication/intervention(s) study procedures and risks will be given to each participant and written documentation of informed consent is required prior to starting study medication/intervention. Participants must sign an informed consent document that has been approved by the REB prior to any procedures being done specifically for the trial. Each participant should have sufficient opportunity to discuss the study, have all of their questions addressed and consider the information in the consent process prior to agreeing to participate. Participants may withdraw consent at any time during the course of the study without prejudice. The informed consent form will be signed and dated by the participant and the qualified investigator (or delegate). The original signed informed consent form will be retained in the participant’s study files and a copy will be provided to the participant.

The informed consent process must be conducted and form signed before the participant undergoes any screening procedures that are performed solely for the purpose of determining eligibility for the study.

# Confidentiality

All participant-related information including Case Report Forms, laboratory specimens, evaluation forms, reports, etc. will be kept strictly confidential. All records will be kept in a secure, locked location and only accessible to research staff. Participants will be identified only by means of a coded number specific to each participant (refer to Section [5](#_bookmark28)). All computerized databases will identify participants by numeric codes only and will be password protected.

Upon request, and in the presence of the investigator or his/her representative, participant records will be made available to the study sponsor, monitoring groups representative of the study sponsor, representatives of funding groups, the research ethics board and applicable regulatory agencies for the purpose of verification of clinical trial procedures and/or data, as is permissible by local regulations.

# Research Ethics Board

The Hamilton Integrated Research Ethics Board (HiREB) will review all appropriate study documentation to safeguard the rights, safety, and well-being of the participants. The study will be conducted only at the site where ethics approval has been obtained. A copy of the protocol (including protocol amendments), all versions of informed consent forms, other information to be completed by participants such as survey instruments or questionnaires, and any proposed advertising/ recruitment materials must be reviewed and approved by the REB prior to implementation of the trial. The investigator will be responsible for obtaining REB approval of the annual Continuing Review throughout the duration of the study. The investigator will notify the REB of serious adverse events. The investigator will seek prior ethics approval for any protocol deviations except when the change intended to eliminate an immediate hazard to participants. In this case, the protocol deviation will be promptly reported.

# General Trial Conduct Considerations

# Adherence to Protocol

# Protocol Amendments

All protocol amendments will be reviewed and approved and if applicable submitted to the applicable regulatory agencies for prior approval or notification. The Investigator must sign and date the amendment prior to implementation. All protocol amendments must also be submitted to the ethics committee.

# Protocol Deviations

No deviations from this protocol will be permitted without the prior written approval of the Investigators, except when the modification is needed to eliminate an immediate hazard or hazards to participants. Any deviations that may affect a participant’s treatment or informed consent, especially those increasing potential risks, must receive prior approval from the REB unless performed to remove an immediate safety risk to the participants. In this case it will be reported to the REB and the Investigators immediately thereafter. Any departures from the protocol must be documented.

# Monitoring & Auditing

# Data Safety and Monitoring Committee (DSMC)

The study will be monitored by the CTN’s Data Safety and Monitoring Committee. The DSMC consists of experts in HIV clinical care, research design, biostatistics and ethics. The DSMC is independent from the investigators and the clinical trial site. The DSMC’s primary responsibilities will be to monitor participant safety and enrolment. The DSMC will meet at specified intervals and at least 4 weeks following the last subject enrolled. The DSMC will review individual and cumulative data for evidence of study-related AE’s and all SAE’s. In conjunction with the sponsor and principal investigators, the DSMC can recommend changes in the protocol and procedures and stopping the trial due to safety concerns or lack of enrolment.

The Investigator must provide the DSMC with:

- - - 1. Prompt reports of any SAEs occurring during the study, to include a written report of events, outcomes of the SAE and relationship to the intervention.
      2. Regular reports of AEs, including cumulative reports 4 weeks after the last subject has completed the intervention.
      3. Any protocol amendments, informed consent changes or revisions of other documents originally submitted for review.
      4. Any new information that may affect adversely the safety of the subjects or the conduct of the study.

# Study Monitoring

The study site agrees to allow monitors from the Sponsor-Investigator and/or their representatives, direct access to the study records, the medical records of participants enrolled in the clinical study, and to drug accountability records. Adequate monitoring space and time must be provided. The Sponsor-Investigator representative will perform study site monitoring after at least the first 2 participants have been enrolled and at regular intervals throughout the study.

The monitor will carry out a quality control of trial progress; including protocol and operating guidelines, data collection, signature of consent forms, completion of SAE reporting, treatment management and sample collection and CRF completion. The Monitor will discuss any problem with the investigator and define the actions to be taken. Close-out activities will be performed at the end of the trial.

# Early Termination of the Trial

As part of their duties to ensure that research participants are protected, the REB or other government organizations may discontinue the study at any time. Regulatory authorities and the Sponsor-Investigator retain the authority to suspend additional enrollment for the entire study as applicable.

The trial may be terminated early on the advice of the independent DSMC based on their review of AE and SAE. The trial may also be terminated early due to low enrollment.

# Record Keeping

# Data Collection

Data for this study will be recorded in the participant’s chart and entered into the study specific electronic case report forms (eCRFs).

# Data Corrections

Corrections of data entered on source documents / data collection worksheets must be entered into the study eCRFs. Data corrections in the eCRFs will be automatically tracked.

# Source Documents

The Investigator must maintain adequate and accurate source documents upon which CRFs for each participant are based. They are to be separate and distinct from CRFs except for cases in which the Sponsor has pre-determined where direct data entry into specified pages of the participant’s CRF is appropriate. These records should include detailed notes on:

- - - - Oral and written communication with participant regarding the study treatment (risks/benefits)
      - Participation in trial and signed and dated informed consent forms
      - Inclusion and exclusion criteria details
      - Visit dates
      - Adverse events and concomitant medication
      - Results of relevant examinations
      - Laboratory printouts
      - Participant’s exposure to any concomitant therapy (start/stop dates, dosing details)
      - Reason for premature discontinuation (if applicable)
      - Enrollment number
      - Methods of contraception
      - Compliance/noncompliance protocol deviation information

# Data Management

Instructions concerning the recording of study data on eCRFs will be provided by the CTN Data Management group in the form of a Database User Manual. The study site is responsible for submitting the data in a timely fashion. All data will be maintained on secure computer servers by the CTN.

It is the responsibility of the CTN Data Management group to assure the quality of computerized data for this study. This role extends from protocol development to generation of the final study databases. Detailed aspects of data handling will be described in the Data Management Plan.

# Record Retention

The Investigator will maintain all study records according to the ICH-GCP and applicable regulatory requirement(s). Records will be retained for 25 years, in accordance with applicable regulatory requirement(s). After this time the documents will be destroyed according to policies available at the time. If the Investigator withdraws from the responsibility of keeping the study records, custody must be transferred to a person willing to accept the responsibility.

# Disclosure and publication Policy

- The results of this study will be published in peer-reviewed publications within a reasonable time of its completion.
- The first author will be the individual who has made the most important contribution to the publication and may or may not be the trial principal investigators.
- Additional authors will be those who have made a significant contribution to the overall success of the study.

# REFERENCES

Aagaard, K., Riehle, K., Ma, J., Segata, N., Mistretta, T.A., Coarfa, C., Raza, S., Rosenbaum, S., Van den Veyver, I., Milosavljevic, A.*, et al.* (2012). A metagenomic approach to characterization of the vaginal microbiome signature in pregnancy. PLoS One *7*, e36466.

Barentsen, R., van de Weijer, P.H., and Schram, J.H. (1997). Continuous low dose estradiol released from a vaginal ring versus estriol vaginal cream for urogenital atrophy. European journal of obstetrics, gynecology, and reproductive biology *71*, 73-80.

Bartram, A.K., Lynch, M.D., Stearns, J.C., Moreno-Hagelsieb, G., and Neufeld, J.D. (2011). Generation of multimillion-sequence 16S rRNA gene libraries from complex microbial communities by assembling paired-end illumina reads. Appl Environ Microbiol *77*, 3846-3852.

Bisanz, J.E., Seney, S., McMillan, A., Vongsa, R., Koenig, D., Wong, L., Dvoracek, B., Gloor, G.B., Sumarah, M., Ford, B.*, et al.* (2014). A systems biology approach investigating the effect of probiotics on the vaginal microbiome and host responses in a double blind, placebo-controlled clinical trial of post-menopausal women. PLoS One *9*, e104511.

Brotman, R.M., Ravel, J., Bavoil, P.M., Gravitt, P.E., and Ghanem, K.G. (2014). Microbiome, sex hormones, and immune responses in the reproductive tract: challenges for vaccine development against sexually transmitted infections. Vaccine *32*, 1543-1552.

Buve, A., Jespers, V., Crucitti, T., and Fichorova, R.N. (2014). The vaginal microbiota and susceptibility to HIV. AIDS *28*, 2333-2344.

Canada, Government of (2013). Section 4-8: Canadian Guidelines on Sexually Transmitted Infections – Management and treatment of specific syndromes – Vaginal discharge.

Caporaso, J.G., Kuczynski, J., Stombaugh, J., Bittinger, K., Bushman, F.D., Costello, E.K., Fierer, N., Pena, A.G., Goodrich, J.K., Gordon, J.I.*, et al.* (2010). QIIME allows analysis of high-throughput community sequencing data. Nat Methods *7*, 335-336.

Courant, F., Aksglaede, L., Antignac, J.-P., Monteau, F., Sorensen, K., Andersson, A.-M., Skakkebaek, N.E., Juul, A., and Bizec, B.L. (2010). Assessment of circulating sex steroid levels in prepubertal and pubertal boys and girls by a novel ultrasensitive gas chromatography-tandem mass spectrometry method. The Journal of clinical endocrinology and metabolism *95*, 82-92.

DeSantis, T.Z., Hugenholtz, P., Larsen, N., Rojas, M., Brodie, E.L., Keller, K., Huber, T., Dalevi, D., Hu, P., and Andersen, G.L. (2006). Greengenes, a chimera-checked 16S rRNA gene database and workbench compatible with ARB. Appl Environ Microbiol *72*, 5069-5072.

Dols, J.A., Reid, G., Kort, R., Schuren, F.H., Tempelman, H., Bontekoe, T.R., Korporaal, H., Van der Veer, E.M., Smit, P.W., and Boon, M.E. (2012). PCR-based identification of eight Lactobacillus species and 18 hr-HPV genotypes in fixed cervical samples of South African women at risk of HIV and BV. Diagn Cytopathol *40*, 472-477.

Dragonas, C., Maltaris, T., Binder, H., Kat, M., Mueller, A., Cupisti, S., Hoffmann, I., Beckmann, M.W., and Dittrich, R. (2007). Progesterone bioavailability with a progesterone-releasing silicone vaginal ring in IVF candidates. European journal of medical research *12*, 264-267.

Eriksen, B. (1999). A randomized, open, parallel-group study on the preventive effect of an estradiol-releasing vaginal ring (Estring) on recurrent urinary tract infections in postmenopausal women. American journal of obstetrics and gynecology *180*, 1072-1079.

Ferreira, V.H., Kafka, J.K., and Kaushic, C. (2014). Influence of common mucosal co- factors on HIV infection in the female genital tract. Am J Reprod Immunol *71*, 543-554.

Gabrielsson, J., Wallenbeck, I., and Birgerson, L. (1996). Pharmacokinetic data on estradiol in light of the estring concept. Estradiol and estring pharmacokinetics. Acta Obstet Gynecol Scand Suppl *163*, 26-31; discussion 32-24.

Goldman, R.D. (2013). Child health update: estrogen cream for labial adhesion in girls. Canadian family physician Medecin de famille canadien *59*, 37-38.

Grisendi, V., Spada, E., Argento, C., Plebani, M., Milani, S., Seracchioli, R., Volpe, A., and La Marca, A. (2014). Age-specific reference values for serum FSH and estradiol levels throughout the reproductive period. Gynecological Endocrinology *30*, 451-455.

Henriksson, L., Stjernquist, M., Boquist, L., Cedergren, I., and Selinus, I. (1996). A one- year multicenter study of efficacy and safety of a continuous, low-dose, estradiol- releasing vaginal ring (Estring) in postmenopausal women with symptoms and signs of urogenital aging. American journal of obstetrics and gynecology *174*, 85-92.

Huhtinen, K., Desai, R., Ståhle, M., Salminen, A., Handelsman, D.J., Perheentupa, A., and Poutanen, M. (2012). Endometrial and endometriotic concentrations of estrone and estradiol are determined by local metabolism rather than circulating levels. The Journal of clinical endocrinology and metabolism *97*, 4228-4235.

Hummelen, R., Changalucha, J., Butamanya, N.L., Cook, A., Habbema, J.D., and Reid,

G. (2010a). Lactobacillus rhamnosus GR-1 and L. reuteri RC-14 to prevent or cure bacterial vaginosis among women with HIV. Int J Gynaecol Obstet *111*, 245-248.

Hummelen, R., Vos, A.P., van't Land, B., van Norren, K., and Reid, G. (2010b). Altered host-microbe interaction in HIV: a target for intervention with pro- and prebiotics. Int Rev Immunol *29*, 485-513.

Janfaza, M., Sherman, T.I., Larmore, K.A., Brown-Dawson, J., and Klein, K.O. (2006). Estradiol levels and secretory dynamics in normal girls and boys as determined by an ultrasensitive bioassay: a 10 year experience. Journal of pediatric endocrinology & metabolism : JPEM *19*, 901-909.

Joag, V.R., McKinnon, L.R., Liu, J., Kidane, S.T., Yudin, M.H., Nyanga, B., Kimwaki, S., Besel, K.E., Obila, J.O., Huibner, S.*, et al.* (2016). Identification of preferential CD4+ T- cell targets for HIV infection in the cervix. Mucosal Immunol *9*, 1-12.

Karimi, K., Inman, M.D., Bienenstock, J., and Forsythe, P. (2009). Lactobacillus reuteri- induced regulatory T cells protect against an allergic airway response in mice. Am J Respir Crit Care Med *179*, 186-193.

Kaushic, C., Roth, K.L., Anipindi, V., and Xiu, F. (2011). Increased prevalence of sexually transmitted viral infections in women: the role of female sex hormones in regulating susceptibility and immune responses. J Reprod Immunol *88*, 204-209.

Kenigsberg, L., Balachandar, S., Prasad, K., and Shah, B. (2013). Exogenous pubertal induction by oral versus transdermal estrogen therapy. Journal of pediatric and adolescent gynecology *26*, 71-79.

Kenyon, C., Colebunders, R., Crucitti, T., Koumans, E.H., Sternberg, M., Bruce, C., al, e., Klebanoff, M.A., Schwebke, J.R., Zhang, J.*, et al.* (2013). The global epidemiology of bacterial vaginosis: a systematic review. American Journal of Obstetrics and Gynecology *209*, 505-523.

Kruse, A.J., van Melick, M., and Bourdrez, P. (2010). A chance finding: preoperative imaging of the urogenital tract proved very useful. Am J Obstet Gynecol *202*, 95 e91-92.

Kruse, G.R., Barbour, R., Heimer, R., Shaboltas, A.V., Toussova, O.V., Hoffman, I.F., and Kozlov, A.P. (2009). Drug choice, spatial distribution, HIV risk, and HIV prevalence among injection drug users in St. Petersburg, Russia. Harm Reduct J *6*, 22.

Kumetz, L.M., Quint, E.H., Fisseha, S., and Smith, Y.R. (2006). Estrogen treatment success in recurrent and persistent labial agglutination. Journal of pediatric and adolescent gynecology *19*, 381-384.

Lajoie, J., Juno, J., Burgener, A., Rahman, S., Mogk, K., Wachihi, C., Mwanjewe, J., Plummer, F.A., Kimani, J., Ball, T.B., and Fowke, K.R. (2012). A distinct cytokine and chemokine profile at the genital mucosa is associated with HIV-1 protection among HIV- exposed seronegative commercial sex workers. Mucosal Immunol *5*, 277-287.

Lozupone, C., Lladser, M.E., Knights, D., Stombaugh, J., and Knight, R. (2011). UniFrac: an effective distance metric for microbial community comparison. ISME J *5*, 169-172.

Martin, H.L., Richardson, B.A., Nyange, P.M., Lavreys, L., Hillier, S.L., Chohan, B., Mandaliya, K., Ndinya-Achola, J.O., Bwayo, J., and Kreiss, J. (1999). Vaginal lactobacilli, microbial flora, and risk of human immunodeficiency virus type 1 and sexually transmitted disease acquisition. The Journal of infectious diseases *180*, 1863-1868.

Martin, M. (2011). Cutadapt removes adapter sequences from high-throughput sequencing reads. EMBnet Journal *17*, 10-12.

Maruoka, R., Tanabe, A., Watanabe, A., Nakamura, K., Ashihara, K., Tanaka, T., Terai, Y., and Ohmichi, M. (2014). Ovarian estradiol production and lipid metabolism in postmenopausal women. Menopause *21*, 1129-1135.

Masella, A.P., Bartram, A.K., Truszkowski, J.M., Brown, D.G., and Neufeld, J.D. (2012). PANDAseq: paired-end assembler for illumina sequences. BMC Bioinformatics *13*, 31. Mastromarino, P., Vitali, B., and Mosca, L. (2013). Bacterial vaginosis: a review on clinical trials with probiotics. New Microbiol *36*, 229-238.

Mayoglou, L., Dulabon, L., Martin-Alguacil, N., Pfaff, D., and Schober, J. (2009). Success of Treatment Modalities for Labial Fusion: A Retrospective Evaluation of Topical and Surgical Treatments. Journal of Pediatric and Adolescent Gynecology *22*, 247-250.

McDonald, D., Price, M.N., Goodrich, J., Nawrocki, E.P., DeSantis, T.Z., Probst, A., Andersen, G.L., Knight, R., and Hugenholtz, P. (2012). An improved Greengenes taxonomy with explicit ranks for ecological and evolutionary analyses of bacteria and archaea. ISME J *6*, 610-618.

McMurdie, P.J., and Holmes, S. (2013). phyloseq: an R package for reproducible interactive analysis and graphics of microbiome census data. PLoS One *8*, e61217.

Mirmonsef, P., Hotton, A.L., Gilbert, D., Burgad, D., Landay, A., Weber, K.M., Cohen, M., Ravel, J., and Spear, G.T. (2014). Free Glycogen in Vaginal Fluids Is Associated with Lactobacillus Colonization and Low Vaginal pH. PLoS One *9*, e102467-e102467.

Mishell, D.R., Thorneycroft, I.H., Nakamura, R.M., Nagata, Y., and Stone, S.C. (1972). Serum estradiol in women ingesting combination oral contraceptive steroids. American journal of obstetrics and gynecology *114*, 923-928.

Muyzer, G., de Waal, E.C., and Uitterlinden, A.G. (1993). Profiling of complex microbial populations by denaturing gradient gel electrophoresis analysis of polymerase chain reaction-amplified genes coding for 16S rRNA. Appl Environ Microbiol *59*, 695-700.

Naessen, T., and Rodriguez-Macias, K. (2002). Endometrial thickness and uterine diameter not affected by ultralow doses of 17beta-estradiol in elderly women. Am J Obstet Gynecol *186*, 944-947.

Naessen, T., Rodriguez-Macias, K., and Lithell, H. (2001). Serum Lipid Profile Improved by Ultra-Low Doses of 17β-Estradiol in Elderly Women <sup>1</sup>. The Journal of Clinical Endocrinology & Metabolism *86*, 2757-2762.

Ravel, J., Gajer, P., Abdo, Z., Schneider, G.M., Koenig, S.S.K., McCulle, S.L., Karlebach, S., Gorle, R., Russell, J., Tacket, C.O.*, et al.* (2011a). Vaginal microbiome of reproductive- age women. Proceedings of the National Academy of Sciences of the United States of America *108 Suppl*, 4680-4687.

Ravel, J., Gajer, P., Abdo, Z., Schneider, G.M., Koenig, S.S.K., McCulle, S.L., Karlebach, S., Gorle, R., Russell, J., Tacket, C.O.*, et al.* (2011b). Vaginal microbiome of reproductive- age women. Proceedings of the National Academy of Sciences of the United States of America, 4680-4687.

Reid, G. (2008). Probiotic Lactobacilli for urogenital health in women. J Clin Gastroenterol

*42 Suppl 3 Pt 2*, S234-236.

Reid, G., Dols, J., and Miller, W. (2009). Targeting the vaginal microbiota with probiotics as a means to counteract infections. Curr Opin Clin Nutr Metab Care *12*, 583-587.

Relman, D.A., Loutit, J.S., Schmidt, T.M., Falkow, S., and Tompkins, L.S. (1990). The agent of bacillary angiomatosis. An approach to the identification of uncultured pathogens. N Engl J Med *323*, 1573-1580.

Schloss, P.D., Westcott, S.L., Ryabin, T., Hall, J.R., Hartmann, M., Hollister, E.B., Lesniewski, R.A., Oakley, B.B., Parks, D.H., Robinson, C.J.*, et al.* (2009). Introducing mothur: open-source, platform-independent, community-supported software for describing and comparing microbial communities. Appl Environ Microbiol *75*, 7537-7541.

Schmidt, G., Andersson, S.B., Nordle, O., Johansson, C.J., and Gunnarsson, P.O. (1994). Release of 17-beta-oestradiol from a vaginal ring in postmenopausal women: pharmacokinetic evaluation. Gynecologic and obstetric investigation *38*, 253-260.

Schober, J., Dulabon, L., Martin-Alguacil, N., Kow, L.-M., and Pfaff, D. (2006). Significance of Topical Estrogens to Labial Fusion and Vaginal Introital Integrity. Journal of Pediatric and Adolescent Gynecology *19*, 337-339.

Sibley, C.D., Grinwis, M.E., Field, T.R., Eshaghurshan, C.S., Faria, M.M., Dowd, S.E., Parkins, M.D., Rabin, H.R., and Surette, M.G. (2011). Culture enriched molecular profiling of the cystic fibrosis airway microbiome. PLoS One *6*, e22702.

Sibley, C.D., Parkins, M.D., Rabin, H.R., Duan, K., Norgaard, J.C., and Surette, M.G. (2008). A polymicrobial perspective of pulmonary infections exposes an enigmatic pathogen in cystic fibrosis patients. Proc Natl Acad Sci U S A *105*, 15070-15075.

Tourgeman, D.E., Gentzchein, E., Stanczyk, F.Z., and Paulson, R.J. (1999). Serum and tissue hormone levels of vaginally and orally administered estradiol. American journal of obstetrics and gynecology *180*, 1480-1483.

Tulchinsky, D., Hobel, C.J., Yeager, E., and Marshall, J.R. (1972). Plasma estrone, estradiol, estriol, progesterone, and 17-hydroxyprogesterone in human pregnancy. I. Normal pregnancy. American Journal of Obstetrics and Gynecology *112*, 1095-1100.

Vujic, G., Jajac Knez, A., Despot Stefanovic, V., Kuzmic Vrbanovic, V. (2013). Efficacy of orally applied probiotic capsules for bacterial vaginosis and other vaginal infections: a double-blind, randomized, placebo-controlled study. Eur J Obstet Gynecol Reprod Biol *168*, 75-79.

Wang, Q., Garrity, G.M., Tiedje, J.M., and Cole, J.R. (2007). Naive Bayesian classifier for rapid assignment of rRNA sequences into the new bacterial taxonomy. Appl Environ Microbiol *73*, 5261-5267.

Weisberg, E., Ayton, R., Darling, G., Farrell, E., Murkies, A., O'Neill, S., Kirkegard, Y., and Fraser, I.S. (2005). Endometrial and vaginal effects of low-dose estradiol delivered by vaginal ring or vaginal tablet. Climacteric *8*, 83-93.

Whelan, F.J., Verschoor, C.P., Stearns, J.C., Rossi, L., Luinstra, K., Loeb, M., Smieja, M., Johnstone, J., Surette, M.G., and Bowdish, D.M. (2014). The loss of topography in the microbial communities of the upper respiratory tract in the elderly. Ann Am Thorac Soc *11*, 513-521.

Wira, C.R., Rodriguez-Garcia, M., and Patel, M.V. (2015). The role of sex hormones in immune protection of the female reproductive tract. Nat Rev Immunol *15*, 217-230.

Ye, Y. (2011). Identification and Quantification of Abundant Species from Pyrosequences of 16S rRNA by Consensus Alignment. Proceedings (IEEE Int Conf Bioinformatics Biomed) *2010*, 153-157.

# APPENDIX

# Study sexual history questionnaire

1. How do you identify your sexual orientation?
   1. Bi-Sexual
   2. Gay
   3. Heterosexual (Straight)
   4. Lesbian
   5. Queer
   6. Questioning
   7. Other: Specify
   8. Not sure
   9. Choose not to respond
2. **In your lifetime,** how many sexual partners have you had vaginal sex with?
   1. 0

**b.** 1-5

**c.** 6-15

**d.** 16-29

1. 30 or more
2. Don’t know
3. Choose not to respond
4. **During the past 6 months**, how many sexual partners have you had vaginal sex with?
   1. 0

**b.** 1-5

**c.** 6-15

**d.** 16-29

1. 30 or more
2. Don’t know
3. Choose not to respond
4. **During the past 6 months,** how often did you use a condom during vaginal sex? ***(Please circle one)***
   1. Never (0%)
   2. Rarely (less than 25% of the time)
   3. Sometimes (25-49% of the time)
   4. Most of the time (50-74% of the time)
   5. Almost every time (75-99% of the time)
   6. All of the time (100% of the time)

# You have finished the questionnaire.

**Again, thank you very much for participating today.**
