# Supplementary material for: Feasibility, safety and tolerability of estrogen and/or probiotics for improving vaginal health in Canadian African, Caribbean, and Black women: A pilot phase 1 clinical trial
Source: PLoS One. 2025 Jan 21;20(1):e0315576. doi: 10.1371/journal.pone.0315576 (PMC11750099; doi:10.1371/journal.pone.0315576)
Supplement: Attachment — Submitted filename: Reply to Reviewers-March 4.docx [file pone.0315576.s004.docx]

**Reply to Reviewers:**

Additional Editor Comments (if provided):

*Please address the comments of the reviewers*

We thank the Editor for their comment and have addressed all the comments made by reviewers as detailed below.

**Reviewer #1:**

*This paper reports the results of a prospective, randomized, open-label phase 1 clinical trial to determine the feasibility, safety, and tolerability of administering low-dose estrogen, probiotics, or both in combination to improve vaginal health and decrease HIV susceptibility. The manuscript if well written, easy to follow and straight forward. I am particularly pleased with the decision to focus enrollment on African, Caribbean, and Black women, who are historically underrepresented in research. I wholeheartedly agree that acceptance of formulation and delivery methods need to be considered when applying probiotics to different body sites and for diverse populations.*

*Comments to address:*

1) *I was a bit disappointed about the lack of biological outcome data in this paper, like for example displacement of anaerobic and BV-associated microbiota, normalizing vaginal pH, and decreasing genital inflammation along with clinical signs and symptoms of BV (it looks like this part will be published separately in the future). Unless there is a rationale for splitting the paper, I would prefer to see a complete story (this paper feels amputated).*

We thank the reviewer for their comments and agree that the biological outcome data is an important aspect of the study. We are in the process of completing this analysis which will include biological outcomes related to the results of the microbiome analysis and the impact of the intervention on genital inflammation. Given the extensive biological data including microbiome analysis, correlation of treatment benefits with microbiome analysis, qPCR and multi-plex cytokine analysis and their correlation to clinical benefits that was conducted, we felt it best to separate the two papers to ensure an in-depth coverage of safety, feasibility and tolerability as well as biological response. The correlation analysis to synthesize clinical benefit is currently being completed, therefore it would be best to publish this first paper first. Combining both sets of data will make the manuscript too lengthy and delay communicating the information on primary outcomes of the Phase I clinical trial.

2) *Other limitations include the small sample size limiting generalization (was acknowledged by the authors), lack of placebo arm (should be acknowledged by the authors).*

We thank the reviewer for their comment and while this phase 1 trial was designed to test the effect of intervention on each participant before and after the treatment, we acknowledge that the inclusion of a placebo arm would be a better study design. We acknowledge that the conclusion of the effect of the intervention in this study design could be confounded by other temporal changes that overlap with the length of intervention, such as diet, seasonal and other changes. The fourth paragraph of the discussion (lines 450-453) has been updated to acknowledge the lack of placebo arm as a limitation due to the availability of resources. In addition, we have made note of the inclusion of a placebo arm for future studies.

3) *I was also surprised by the number of adverse events. Are those severe systemic effects (like nausea, light headedness, headache, cramps) expected with this type of intervention? It is hard to evaluate without a placebo arm.*

We appreciate the reviewer’s insights and concern. The adverse events reported by participants including nausea, light headedness, headache, and cramps were not surprising with this type of intervention, since these are listed as side effects of RepHresh™ Pro-B™ and/or Estring©. The study protocol (Protocol S1) included with the supplemental materials provides a detailed overview of the expected adverse events based on previous studies and manufacturer details. The second paragraph (Lines 418-421) of the discussion quoted below, highlights these adverse events as the most commonly reported side effects for these study products, with multiple references included. “Consistent with the safety profile of *Lactobacillus*-based probiotics including those reported specifically for *L. rhamnosus* GR-1 and *L. reuteri* RC-14, the most common local side effects were cramps/abdominal pain and vaginal discomfort which were mild and infrequent (25,30,31,34,50). Similarly, the most common AEs included headaches and increased vaginal secretions for Estring© users (41,51-53).” Of note, these effects were temporary and almost all resolved within a short time. Importantly the side effects did not have any impact on the acceptability and adherence of the intervention.

4) *Importantly, do NOT use the word subject when referring to study participants (it is stigmatizing and not acceptable).*

We thank the reviewer for highlighting this issue. We agree that participant is much better terminology than subjects and apologies for this oversight. The manuscript has been updated accordingly and no longer uses the term “subject(s)” to refer to study participants. The four instances in which this was done have now been changed to “participant(s)”, shown in track changes in the revised manuscript.

**Reviewer #2:**

*This paper summarizes the safety, feasibility, and tolerability of innovative intervention regimens to improve vaginal health. It was a randomized phase 1 open label in which participants were randomized to one of 4 arms: Intravaginal estradiol (estring 7.5mg/day), vaginal probiotic 2x/day, estradiol + vaginal, estradiol + oral twice daily. Interventions were taken for 30 days. There was high and comparable adherence across arms. While the AE rate was high, they were primarily mild and resolved by end of study, with no SAEs. The results are sufficient to move forward with larger trials of these potential interventions. The paper is nicely written, and only minor clarifications are requested.*

*Comments to address:*

1) *Could the authors please clarify what is the unit on adherence? E.g., is 0.93 --> 93% of doses were taken?*

We thank the reviewer for their comment regarding the adherence units. We have updated the manuscript to list adherence metrics as a percentage to improve readability. The specific definitions describing how adherence values were determined are described in the “Outcome Measures” section in the Materials and Methods (lines 238-240).

2) *There seems to be a jump in rationale explaining the selection of Lacticacseibacillus rhamnosus and Limosillactobacillus reuteri (lines 92-97). Could the authors please add a sentence or two about how these strains address the sentence (92-94), or the “properties” they are addressing in line 94? Reviewer notes later there are some mechanisms at lines 111-113 that may flow better above.*

We thank the reviewer for their feedback. We have updated the introduction to more clearly address the properties mentioned on line 92-94 by taking the reviewer’s suggestion and moving the description of the mechanisms on from next paragrpah to add to previous paragraph (lines 91-96) to improve the flow of this section.

3) *Lines 106-108, could authors clarify here whether the studies citing topical estrogen creams employed safely and well-tolerated were in pre-menopausal women? It may be there in the citations, but just a few words to clarify and save the reader from having to look something up.*

We thank the reviewer for their insight and have included a statement clarifying that the studies cited in the text for both topical creams and vaginal rings containing estrogen were in post-menopausal women, to improve clarity of this section of the manuscript (lines114-116).

4) *Line 109. The use of the word “demonstrate” is confusing in this sentence: “we are testing an innovative strategy”?*

We thank reviewer 2 for their feedback and have taken their suggestion to replace the word “demonstrate” on line 109 with “we are testing an innovative strategy” to improve clarity of this sentence (line 117).

5) *Line 135. Could the authors please specify the amount of compensation?*

We thank reviewer 2 for their comment and have included the amount of compensation ($100/visit) in the Material and Methods. Additional details regarding the incentive referral process can be found in the detailed study protocol (Protocol-S1) which is included as a supplemental document.

6) *Lines 145-146. Could the authors please explain at least some of the inclusion and exclusion criteria in the text? To have none there is disruptive to reading. Or make this a table in the main paper (I don’t think Plos limits these?) because it’s disruptive to have to go to supplemental to read this.*

We appreciate reviewer 2’s comment and have moved the inclusion and exclusion criteria table from the supplemental information to the main text. This is now listed as Table 1 in the manuscript with a detailed description of the criteria described in the study protocol (S1-Protocol) attached as supplemental material.

7) *Irregular menstrual cycle is listed as an exclusion criterion. Could the authors please explain how this was assessed?*

Thank you for this comment. The irregular menstrual cycle was assessed by the study nurse during the initial screening interview. This is described in the study protocol (Protocol S1) included as supplemental materials. We have updated the methods section as described in the previous comment to now include inclusion/exclusion criteria in Table 1, with a clear reference to the study protocol for further details.

8) *Could the authors please explain why 4 participants withdrew consent?*

We appreciate reviewer 2 inquiring about the participants who withdrew consent. These four participants no longer wished to partake in the study and chose to withdraw between visits 2 and 3. They explained to the study nurse that they had not been able to use the intervention as required in the study protocol and therefore wished to withdraw from the study.

9) *Lines 227-229: Reviewer is confused on what is meant by “prematurely terminated the study” (dropped out? Stopped coming for study visits?) as they are then “considered non-adherent” (line 229) so it would be important to understand why they were non-adherent/prematurely terminated.*

We thank reviewer 2 for their comment and apologize for the confusion. We have now clarified the three categories of participants who were given a value of 0 in the adherence metrics: 1) those who were non-adherent and wished to withdraw consent to participate in the study (n=4) 2) those who were lost to follow up (n=1) and 3) those who were non-adherent (but did not withdraw consent) and did not complete the study (n=4). As described in the following line (lines 240-243) participants who did not complete the intervention protocol (for any of the three reasons above) were assigned a value of 0 when calculating adherence metrics. We have removed the text and removed “premature termination” as it was creating confusion.

10) *Line 239, it is unclear why “congenital anomaly” is listed as a potential SAE of this intervention?*

We thank reviewer 2 for their comment. The inclusion of “congenital anomaly” listed as an SAE is based on the definition of Severe Adverse Events. This is a general, all-encompassing definition of SAEs for clinical trials, as per US Department of HHS Guidelines for Grading Severity of Adult and Pediatric Adverse Events and is not specific to this trial. We have referenced the source for this definition in the text on Line 255.

11) *Lines 288-290: Could the authors please clarify the adherence rate units – is tis 92% 93% 97% etc.? Are these adherence rates for all 50 women who were randomized or just those n=41 completing? It seems like it is for all based implication in lines 292-293. But this is somewhat confusing b/c the CONSORT diagram indicates that these “participants terminating study prematurely” (line 293) are not included in analyses. But then the safety markers are also analyzed across the 50 participants (368)*

We thank reviewer 2 for their comment. The adherence rate units are indeed same as % and have been altered in the revised manuscript and associated Figure 5. Reviewer 2 is correct that the adherence rates are determined based on all 50 women who received the intervention. The text on Lines 304-308 has been updated to better describe how adherence was determined and which participants were included in the calculation.

12) *Kudos to investigators on the high adherence and completion rates! Were participants provided any incentives or reminders?*

We thank reviewer 2 for their positive feedback. Participants were provided with compensation for their time and additional expenses and the nurse sent text/phone call reminders of clinic appointments. The details for the compensation/incentives can be found in the study protocol (Protocol S1).

13) *As with comment above, wording in lines 416-418 is confusing use of “demonstrate” – “we demonstrate an innovative strategy”. “we evaluate? Test? Examine?”*

We thank reviewer 2 for their comment and have updated the wording to avoid the use of the word “demonstrate” in this sentence. We have replaced “demonstrate” with “evaluate” to improve clarity and readability (Line 434).

14) *Lines 421-422 – it seems something got cut out of this sentence. In what ways “more beneficial” for?*

We thank reviewer 2 for making note of this oversight. We have updated this sentence to reflect that the “intervention may be more beneficial for ACB women where *L. crispatus* may not be the predominant microbial species present within the vaginal microbiome”. (Lines 439-440)

**Editorial Comments for Journal Guidelines:**

1) *Please ensure that your manuscript meets PLOS ONE's style requirements, including those for file naming.*

The manuscript meets the PLOS ONE style requirements including those described for file naming.

2) *Thank you for stating in your Funding Statement:*

*“This study was funded by CIHR Grants FRN#159229 and FRN#154047 (CK) and in kind contributions from CIHR HIV Clinical Trials Network (FS, CK).*

*B.G was partially supported by a Post-doctoral Award from CIHR FRN#181875”*

*Please provide an amended statement that declares *all* the funding or sources of support (whether external or internal to your organization) received during this study, as detailed online in our guide for authors at http://journals.plos.org/plosone/s/submit-now. Please also include the statement “There was no additional external funding received for this study.” in your updated Funding Statement.*

The statement has been changed in the manuscript text.

*Please include your amended Funding Statement within your cover letter. We will change the online submission form on your behalf.*

We have included this in the cover letter. Thank you for changing it online.

3) *Thank you for stating the following in the Competing Interests section:*

*“I have read the journal's policy and the authors of this manuscript have the following competing interests: GR developed the probiotic strains GR-1 and RC-14 but has had no financial interest in them for 15 years. GR consults for Seed, a company producing probiotic strains not used in this study. All the other authors have declared no conflict of interest.”*

*We note that one or more of the authors are employed by a commercial company: Seed*

Dr. Gregor Reid has clarified that he was not employed by seed but has been involved as an advisor to this company, regardless of the different titles he was given. He has never received any salary from the company.

*a. Please provide an amended Funding Statement declaring this commercial affiliation, as well as a statement regarding the Role of Funders in your study. If the funding organization did not play a role in the study design, data collection and analysis, decision to publish, or preparation of the manuscript and only provided financial support in the form of authors' salaries and/or research materials, please review your statements relating to the author contributions, and ensure you have specifically and accurately indicated the role(s) that these authors had in your study. You can update author roles in the Author Contributions section of the online submission form.*

*Please also include the following statement within your amended Funding Statement.*

*“The funder provided support in the form of salaries for authors [insert relevant initials], but did not have any additional role in the study design, data collection and analysis, decision to publish, or preparation of the manuscript. The specific roles of these authors are articulated in the ‘author contributions’ section.”*

*If your commercial affiliation did play a role in your study, please state and explain this role within your updated Funding Statement.*

Since the company did not provide any salary support for Dr. Reid, there is no need for any changes in the funding statement.

*b. Please also provide an updated Competing Interests Statement declaring this commercial affiliation along with any other relevant declarations relating to employment, consultancy, patents, products in development, or marketed products, etc.*

*Within your Competing Interests Statement, please confirm that this commercial affiliation does not alter your adherence to all PLOS ONE policies on sharing data and materials by including the following statement: "This does not alter our adherence to PLOS ONE policies on sharing data and materials.” (as detailed online in our guide for authors http://journals.plos.org/plosone/s/competing-interests) . If this adherence statement is not accurate and there are restrictions on sharing of data and/or materials, please state these. Please note that we cannot proceed with consideration of your article until this information has been declared.*

*Please include both an updated Funding Statement and Competing Interests Statement in your cover letter. We will change the online submission form on your behalf.*

As outlined above, Seed did not provide any salary support to Dr. Reid and therefore there is no change in the funding statement. The competing interest statement has been altered to clarify this. The updated Funding and Competing Interest Statement have been added to the cover letter.

4) *Thank you for stating the following in the Acknowledgments Section of your manuscript:*

*“This study was supported by CIHR Grants FRN# 159229 and #126019 (C.K.) and by in kind support from the CIHR Canadian HIV Trials Network (CTN 308). B.G was partially supported by a Post-doctoral Award from CIHR FRN#181875.”*

*We note that you have provided additional information within the Acknowledgements Section that is not currently declared in your Funding Statement. Please note that funding information should not appear in the Acknowledgments section or other areas of your manuscript. We will only publish funding information present in the Funding Statement section of the online submission form.*

*Please remove any funding-related text from the manuscript and let us know how you would like to update your Funding Statement. Currently, your Funding Statement reads as follows:*

*“This study was funded by CIHR Grants FRN#159229 and FRN#154047 (CK) and in kind contributions from CIHR HIV Clinical Trials Network (FS, CK).*

*B.G was partially supported by a Post-doctoral Award from CIHR FRN#181875”*

*Please include your amended statements within your cover letter; we will change the online submission form on your behalf.*

This has been added to the cover letter.

5) *Please include captions for your Supporting Information files at the end of your manuscript, and update any in-text citations to match accordingly.*

Captions have been added to the end of the manuscript for the supporting information files and all in-text citations match these file names. Files include a supporting information word document with tables S1-S2, Checklist S1 containing the CONSORT checklist and Protocol S1 consisting of the detailed study protocol.

6) *Please review your reference list to ensure that it is complete and correct. If you have cited papers that have been retracted, please include the rationale for doing so in the manuscript text, or remove these references and replace them with relevant current references. Any changes to the reference list should be mentioned in the rebuttal letter that accompanies your revised manuscript. If you need to cite a retracted article, indicate the article’s retracted status in the References list and also include a citation and full reference for the retraction notice.*

The reference list has been reviewed and we can confirm that it is complete with the correct information included. No retracted papers have been cited and no additional changes have been made.
