# Supplementary material for: Feasibility, safety and tolerability of estrogen and/or probiotics for improving vaginal health in Canadian African, Caribbean, and Black women: A pilot phase 1 clinical trial
Source: PLoS One. 2025 Jan 21;20(1):e0315576. doi: 10.1371/journal.pone.0315576 (PMC11750099; doi:10.1371/journal.pone.0315576)
Supplement: Attachment — Submitted filename: Reply to Reviewers-June 6.docx [file pone.0315576.s005.docx]

**Reply to Reviewers:**

**Reviewer #3:**

*Statistically, this manuscript is pretty straightforward. For phase 1 safety trials, this is usually the case since the purpose and sample size are not conducive for performing more complicated analyses. Your stated goals of the trial in line 114 align with this and, as I see it, assessing "feasibility, safety, and tolerability" are more about estimating proportions and percentages than doing any sort of comparative analyses.

That said, I am not sure about the sample sizes for the arms in this trial. I know there exists literature on calculating the sample size for phase 1 dose-finding trials, but I know less about safety trials. Logically, I would think that the sample size should be large enough to capture AEs (and especially SAEs) that occur with some frequency in the population being studied. For the overall numbers, I think you are ok. That's because, if we assume that the probability of an AE is 0.1, then, using the binomial distribution, the probability that you would not detect that AE in 41 people is 0.01. So, only a 1% chance that it would be missed. On the other hand, if we use a sample size of 13 people, that jumps to 0.25 (and 0.31 for 11 people). That's a pretty high probability and I think is too high for comfort. There are no power analyses reported in this manuscript. I think that is a negative, but that can't be changed retrospectively.

I am struggling the most with PLOS publication criterion number 3 (*[*https://journals.plos.org/plosone/s/criteria-for-publication*](https://journals.plos.org/plosone/s/criteria-for-publication)*) since I think the product-specific sample sizes are too low to be robust. I think the conclusions inferred by product, such as lines 384-386, are too strong given the small sample size. I think a greater focus on the overall results and softening the language around the product-specific conclusions is needed.*

**REPLY**: We thank the reviewer for this insightful comment and appreciate the opportunity to clarify the details of our sample sizes within the intervention arms.

This study was designed as a pilot Phase I clinical trial study with the purpose was to examine the feasibility of recruitment, randomization, retention, assessment procedures, and implementation of an novel intervention.

The reviewer questions how to approach calculating the sample size for a Phase 1 safety study. A pilot study cannot inform the safety testing of a phase 1 study and no sample size calculations based on safety outcomes are recommended. ([10.1016/j.jpsychires.2010.10.008](https://doi.org/10.1016%2Fj.jpsychires.2010.10.008)). Only in an extreme, unfortunate case, where a death occurs or repeated serious adverse events surface, can a pilot study inform the safety of testing an intervention due to the small sample size. However, pilot studies provide an opportunity to implement and examine the feasibility of the adverse event reporting system.

While originally each intervention arm was planned to be 20 participants, the trial was stopped after two years when the goals for feasibility and safety data were met, due to the multiple starts and stops in the trial due to COVID. Given the smaller number in each arm of the intervention, we did not statistical power to analyze each study arm. Instead the AE incidence for the Estring was examined by combining all 3 groups that received this intervention (Estring alone, Estring + oral probiotics, Estring+ vaginal probiotics) and for probiotics also from 3 groups (Vaginal probiotic, Estring + oral probiotics, Estring+ vaginal probiotics). For the Estring intervention arm, the product-specific sample size was 37 at enrollment and 28 at completion, while for the probiotics arm, it was 37 at enrollment and 32 at completion. This adjustment brings the probability of missing an AE closer to 2-5% based on probability of AE as 0.1, as suggested by the reviewer, rather than 25-30%. Additionally, we want to reiterate that despite the smaller sample sizes, there were no signals for any serious adverse events throughout the entirety of our study.  Nevertheless, we acknowledge the importance of exercising caution and have reflected this sentiment in the revised language in the Discussion section, where we state “With the limitation that the overall sample size was small for this trial, Estring and RepHresh Pro-B use for 30 days did not lead to any serious adverse effects, with no clinical safety concerns identified in the trial”

We sincerely appreciate the reviewer’s valuable feedback, which has allowed us to provide a more nuanced discussion of our findings and their implications.

Reviewer: Specific comments:
1. (lines 146-9) Please reference the computer program used for randomization.

REPLY: The acquisition of test products, packaging, randomization were contracted out to BARL (Bay Area Research Logistics) who specialize in clinical supply and logistical management of clinical trials. The randomization code was generated by the statistician in BARL in block sizes of 4 or 8.

2. (Table 4) I believe the overall sample size for Visit 5 is a typo and should read 41 instead of 4.

REPLY: Many thanks to the reviewer for catching this oversight. This has been corrected.
