# Supplementary material for: Feasibility, safety and tolerability of estrogen and/or probiotics for improving vaginal health in Canadian African, Caribbean, and Black women: A pilot phase 1 clinical trial
Source: PLoS One. 2025 Jan 21;20(1):e0315576. doi: 10.1371/journal.pone.0315576 (PMC11750099; doi:10.1371/journal.pone.0315576)
Supplement: Attachment — Submitted filename: Reply to Reviewers-September 22.docx [file pone.0315576.s006.docx]

**Reply to Reviewers:**

Reviewer #3: Thank you for your thorough consideration of my comments. That said, I am still coming to the same conclusions as my previous review. Based on your response, specifically this:

"This study was designed as a pilot Phase I clinical trial study with the purpose was to examine the feasibility of recruitment, randomization, retention, assessment procedures, and implementation of an novel intervention."

safety is not a part of this study's purpose. And yet the title and abstract seem to report safety findings. Demonstration projects are important and I can understand the argument that such a project is needed to assess the feasibility of testing an intervention and data collection systems. But, the way this manuscript is structured it still looks heavily like you are reporting the results of a safety trial and that safety is one of the objectives of this manuscript.

I think it is unreasonable to expect a phase 1 trial to capture an extremely rare event, such as a 1:1000 event. But, I think it is reasonable for a phase 1 trial to capture a 1:100, which I don't see as an extreme case.

Thus, I still feel the sample sizes are not large enough to produce robust results and that criterion #3 (<https://journals.plos.org/plosone/s/criteria-for-publication>) has not been met.

REPLY: We are sorry that the reviewer feels that the manuscript does not meet the criteria for reporting safety. As explained in our previous reply, we did calculate the probability of missing an AE based on the 1:100 (0.01) chance that the reviewer said was acceptable and we showed that based on the sample size of the study, the probability of missing an AE of 1:100 was between 2-5% and not 25-30% as projected by the reviewer. Furthermore, we altered the language in the Discussion and added the language that “*With the limitation that the overall sample size was small for this trial*, Estring and RepHresh Pro-B use for 30 days did not lead to any serious adverse effects, with no clinical safety concerns identified in the trial” . We have now added “2-5% chance of missing 1:100 AE, based on sample size” to the discussion to be transparent about the limitation of safety data based on the sample size in this trial (Page 21, line 386)

REPLY:

Reviewer #4: The authors have addressed all issues, and I believe the manuscript is ready for publication in its current form.

REPLY: We thank the reviewer and really appreciate their comment that the manuscript is ready for publication.

Reviewer #5: This is an important manuscript addressing a key research question – understanding and mitigating modifiable risk factors for HIV transmission in a high-risk population. The introduction adequately lays out the scientific rationale for the feasibility trial and underlying clinical question being discussed, including current data gaps – need for research to better prevent/treat BV in order to reduce biological susceptibility to HIV, and lack of safety data on use of oral probiotics combined with estrogen vaginally for longer than 5 days. The primary and secondary outcomes are clearly defined, and the study design which includes randomization and intention to treat analysis is sound. The methods section is well laid out and use a schedule events make it easy to follow.

A few thing can be further clarified to strengthen the interpretation of results and support the study’s conclusions:

1) Adherence assessment:
- How was adherence measured exactly, in the participants who completed the trial?
- The manuscript states ‘adherence metrics were determined according to either the ratio of probiotics consumed relative to the total dispensed, or the number of days the estring was used relative to the study duration.” – was this all based on self-report, or were there any objective measures of assessing adherence? The manuscript states that participants kept a diary – what exactly did they note? E.g., those using a probiotics twice a day, did they indicate date/time each probiotic was used for the full 30 days, and how was this assessed/graded to give an adherence score of close to 1 (perfect adherence)? It is often difficult for patients to take a daily pill for 30 days, and we expect it would be difficult to do so twice daily for 30 days, so how was this assessed to give the high adherence scores noted? This needs to be justified further

REPLY: Thank you for your comments. In this study, adherence was measured primarily through self-report, as participants maintained a daily diary throughout the trial and secondly by asking the participants to return of unused probiotic capsules and vaginal ring at the end of the study. Specifically, for participants using probiotics, they recorded each dose taken, including the date and time of day (morning and evening), in their diaries. The participants brought the diary with them to the clinic during their visits which was reviewed by the nurse at Visit 3 (Day 14) and Visit 4 (final visit for end of study, Day 30). At the final visit, the diaries, unused study products (Estring and probiotic pills) as well as empty containers were returned to the clinic. Adherence scores were calculated based on these self-reports by comparing the number of doses recorded in the diary against the total number of doses expected to be used over the study period. The number of pills returned was counted to match with diary reports. Regarding the high adherence scores, while we acknowledge that adherence to twice-daily probiotics for 30 days can be challenging, the data were derived from participant-reported diaries, which were reviewed and cross-checked with the number of probiotics dispensed/returned. This method is commonly used for microbicide and probiotic trials (Cohen et al, 2020 NEJM (ref 31), Happel et al, BMC Infec Dis 2020 (ref 47), Montogomery et al, 2012, AIDS Behav (ref 48). However, we recognize that reliance on self-report can have limitations, such as false reporting. This limitation has been further emphasized in the discussion (Page 23, line 427-30).

- Can an addendum of a sample study diary that is filled out be included in the appendix to show an example of ‘good’ adherence (assuming filled out daily), vs non-ideal adherence? What data did the authors use to back the statement “diary completion across all visits and interventions was 100% among those who completed the study,” and can the authors more explicitly address how this method of assessing diary completion gives them confidence that the participants used the capsules twice daily, which then strengthens the safety conclusions of the trial? This is important, because non-optimal adherence affects the safety outcomes which are important for the trial. If the authors cannot confidently state that the intervention was not used twice daily for 30 days, then this should be included in the limitations to inform interpretation of the AEs noted.

REPLY: Thank you for this comment. The diary completion of 100% refers to number of participants who completed the study and returned completed diary (97% including those that discontinued the intervention). It does not imply that there was 100% adherence to the intervention. Once the diaries were returned, the data from the diaries were counted and adherence rate was calculated across different intervention groups and across the whole study. Based on these calculations, the adherence met the 80% threshold we wanted to see in the trial.

Unfortunately, the journal policy does not allow us to attach examples of hand-filled diaries as appendix to the paper due to potential privacy breach. We were told to remove the examples of diaries since journal policy states: “Data sharing should never compromise participant privacy. It is therefore not appropriate to publicly share personally identifiable data on human research participants. Data collected from a small group of participants, vulnerable populations, or private groups should not be shared since that may risk the identification of study participants”. We have attached a couple of example pages with no identifiers at the bottom of this document for the reviewer’s perusal to show optimal adherence versus non-optimal adherence.

Data was collected and tabulated from all diaries and adherence was calculated for each intervention group. Overall, the adherence for Estring from diary data collected from groups 1, 2 and 3 was 97% and probiotic from groups 2, 3 and 4 was 92% (Fig 3 and Table 4). Given the high adherence rate, barring false self reports, we do not think this would affect the safety outcomes.

2) Adverse event evaluation
- What scale was used to grade the AEs, and is it a standardized scale/one used previously in similar studies? What differentiated a mild from moderate AE? If a standardized grading scale was not used, consider noting this as a limitation as it may reduce reproducibility

REPLY: Adverse events were categorized and the intensity for each AE graded according to the guidelines provided by the Division of AIDS (DAIDS, NIH) Adverse Event Grading Table which has been used in numerous clinical trials funded by DAIDS for safety data reporting to maintain accuracy and consistency in the evaluation of AEs. Table for Grading the Severity of Adult and Pediatric Adverse Events V2.1 (<https://rsc.tech-res.com/clinical-research-sites/safety-reporting/daids-grading-tables> ). This was cited in the Outcome measures section of Methods (page 13, line 232, ref 45). The details of the source of grading scale have not been added into the results section as well (page 19, line 336). Given the standardized scale which has been used widely for other clinical trials, we do not believe this should be added as a limitation.

The DAIDS grading table provides an AE severity grading scale ranging from grades 1 to 5 with descriptions for each AE based on the following general guidelines:

• Grade 1 indicates a mild event

• Grade 2 indicates a moderate event

• Grade 3 indicates a severe event

• Grade 4 indicates a potentially life-threatening event

• Grade 5 indicates death

3) Completion of sexual history questionnaire as adherence assessments
- Can the authors please clarify how completion of the sexual history questionnaire is used as an adherence assessment method? What is the rationale for this? Has this been validated previously or used in similar studies?

REPLY: Thank you for this comment. The sexual history questionnaire was not used for assessment of adherence, but rather for feasibility (page 12, line 222). Sexual history was collected primarily for the biological correlates of microbiota analysis, which was related to the secondary goal of the trial. Sexual intercourse can significantly influence the composition of the vaginal microbiota. Understanding participants' sexual behaviors allowed us to control for potential confounding factors that could affect the microbiota composition. Tracking sexual behaviors provided a way to ensure that participants adhered to the guidelines, which could otherwise influence the microbiota findings. The sexual history questionnaire was not used directly for assessment of adherence described in the results here.

**Optimal adherence example**

**Non-optimal adherence example**
