# Supplementary material for: Feasibility, safety and tolerability of estrogen and/or probiotics for improving vaginal health in Canadian African, Caribbean, and Black women: A pilot phase 1 clinical trial
Source: PLoS One. 2025 Jan 21;20(1):e0315576. doi: 10.1371/journal.pone.0315576 (PMC11750099; doi:10.1371/journal.pone.0315576)
Supplement: Attachment — Submitted filename: Reply to Reviewers-Nov 4.docx [file pone.0315576.s007.docx]

**Reply to Reviewers:**

Reviewer #3: I think I understand better where you are coming from, but I still believe the statement you have added, "2-5% chance of missing 1:100 AE, based on sample size" is not correct.

In lines 334-366, the numbers reported there are all based on N=29. Assuming an event with a probability of 0.001 or 0.1%, we can calculate the probability of observing exactly zero events in 29 people using the binomial distribution. I'm using R.

> pbinom(0,29,0.001)
[1] 0.9714024

That's a 97% chance of observing exactly zero events, i.e., a 97% chance of missing that event. I'm ok with that because I think it is unreasonable to expect a phase 1 trial to detect such a rare event. Using the same approach, we can see what the probability is of observing exactly zero events when the probability is 0.01:

> pbinom(0,29,0.01)
[1] 0.7471721

That says there is approximately a 75% chance of missing an event, which is far greater than the 2-5% in your statement. If you want to get down to your 2-5%, you need to up the probability by an order of magnitude:

> pbinom(0,29,0.10)
[1] 0.04710129

Thus, you can feel confident about observing an AE in your trial if it occurs in approximately 10% of people.

My contention is that is not good enough. I suspect different fields have different standards, so maybe standards are lower in your field. For instance, I can find a paper that suggests sample sizes have been increasing over time (<https://doi.org/10.1093/jnci/dju163>) and that your sample size would be on the lower end for studies performed approximately 35 years ago. But maybe those trials are different and there are differences between your field and theirs.

REPLY:

We thank the reviewer for further elaborating on their previous concerns.

The AE calculations were done on sample size of 37 for each intervention (13+11+13 for estring and 11+13+13 for probiotics, since each intervention was used in 3 out of 4 groups) and not 29. We apologize for the confusion; the number 29 was actually the number of participants who reported AE out of 37. This has been changed and clarified in the manuscript now (Line 334, 337)

Although the sample size of 37 is larger than 29, we assume that this will not address the concern the reviewer has raised because change in sample size from 29 to 37 will not change the detection limit of AE by a log fold. Thank you for acknowledging that it is unreasonable to expect a Phase I trial to detect a rare event. We agree that this is pilot Phase I and it was not designed to detect rare adverse events.

Based on reviewer’s comments regarding differences in standards for different fields, we searched some recent Phase I clinical trials in microbiome area to do some comparisons of what is acceptable in this area and found that sample sizes for Phase I clinical trial were typically between 12-33 participants for different trials and spread over multiple study arms. So, our sample size of 43 is very comparable.

We also found a 2018 FDA document on design of clinical trials as part of drug design process (<https://www.fda.gov/patients/drug-development-process/step-3-clinical-research#Clinical_Research_Phase_Studies>), which provides typical sample sizes and description for different phases of clinical trials. Below we provide the text from the FDA website, describing Phase I clinical trial.

**“Study Participants:** 20 to 100 healthy volunteers or people with the disease/condition.

**Length of Study:** Several months

[**Purpose:** Safety and dosage](https://www.fda.gov/patients/drug-development-process/step-3-clinical-research#collapse1)

During Phase 1 studies, researchers test a new drug in normal volunteers (healthy people). In most cases, 20 to 80 healthy volunteers or people with the disease/condition participate in Phase 1. However, if a new drug is intended for use in cancer patients, researchers conduct Phase 1 studies in patients with that type of cancer.

Phase 1 studies are closely monitored and gather information about how a drug interacts with the human body. Researchers adjust dosing schemes based on animal data to find out how much of a drug the body can tolerate and what its acute side effects are.

As a Phase 1 trial continues, researchers answer research questions related to how it works in the body, the side effects associated with increased dosage, and early information about how effective it is to determine how best to administer the drug to limit risks and maximize possible benefits. This is important to the design of Phase 2 studies.

Approximately 70% of drugs move to the next phase**”**

Again, our sample size of 43 falls within the range of the description provided above.

We outline below some examples we found of the Phase I clinical trials in the microbiome area

1. Phase 1 clinical trial evaluating safety, bioavailability, and gut microbiome with a combination of curcumin and ursolic acid in lipid enhanced capsules. [Journal of Traditional and Complementary Medicine](https://www.sciencedirect.com/journal/journal-of-traditional-and-complementary-medicine), 2024.

**Sample size: 18 subjects**

<https://doi.org/10.1016/j.jtcme.2024.03.002>

2. Multiple-Ascending-Dose Phase 1 Clinical Study of the Safety, Tolerability, and Pharmacokinetics of CRS3123, a Narrow-Spectrum Agent with Minimal Disruption of Normal Gut Microbiota **A**ntimicrob Agents Chemother 2019 Dec 20;64(1):e01395-19.

**Sample size: 30 subjects in 3 cohorts of 10 each**

doi: [10.1128/AAC.01395-19](https://doi.org/10.1128/AAC.01395-19)

3. A phase 1, randomised, double-blind, placebo-controlled, study to evaluate the safety, tolerability and pharmacokinetics of MAP 315 in healthy adults <https://microba.com/news/successful-ibd-phase-i-clinical-trial/> (<https://www.anzctr.org.au/Trial/Registration/TrialReview.aspx?id=385380>)

**Sample size: 2 cohorts of 16 participants each**

4. Randomized Placebo-Controlled, Biomarker-Stratified Phase Ib Microbiome Modulation in Melanoma: Impact of Antibiotic Preconditioning on Microbiome and Immunity

***Cancer Discov* (2024) 14 (7): 1161–1175.**

**Sample size: 14 patients in 2 arms**

[**https://doi.org/10.1158/2159-8290.CD-24-0066**](https://doi.org/10.1158/2159-8290.CD-24-0066)

A couple of specific examples of Phase I trials to test interventions for *vaginal microbiome*

1.Phase 1 Dose-ranging Safety Trial of *Lactobacillus crispatus* CTV-05 (LACTIN-V) for the Prevention of Bacterial Vaginosis. Sex Trans Dis. 2010

**Sample size: 12 volunteers in 3 blocks of four**

<https://pmc.ncbi.nlm.nih.gov/articles/PMC2758081/>

Below the text from the results of this paper describing Adverse events:

**Adverse events**

By definition AEs occurred any time after the first applicator was administered and within 35 days of enrollment. All 12 randomized subjects (100%) reported at least one AE. AEs reported in two or more subjects included: vaginal discharge (5 subjects, 42%), abdominal pain (4 subjects, 33%), metrorrhagia (4 subjects, 33%), vulvovaginitis (4 subjects, 33%), vaginal candidiasis (3 subjects, 25%), vaginal odor (3 subjects, 25%) and urinary tract infection (2 subjects, 17%). AEs were mostly mild in severity (41 out of 45 total AEs, 91%) and occurred among 11 subjects. Four of 45 total AEs (9%) were moderate in severity, occurred among 4 subjects and were determined to be unrelated or probably unrelated to study product: in the LACTIN-V arm one woman suffered from gastroenteritis and another woman had a urinary tract infection while in the placebo arm one woman reported ear pain and another woman experienced an upper respiratory tract infection ([Table 1](https://pmc.ncbi.nlm.nih.gov/articles/PMC2758081/#T1)). One unexpected AE occurred at the highest dose when an applicator tip separated from the barrel during product administration; this event was not associated with any clinical signs or symptoms. No grade 3 or 4 severity AEs or SAEs occurred during the study.

2.Comparative phase I randomized open-label pilot clinical trial of Gynophilus^®^ (Lcr regenerans^®^) immediate release capsules versus slow release muco-adhesive tablets

Eur J Clin Microbiol Infect Dis 2018 Jul 21;37(10):1869–1880**.**

**Sample size: 33 volunteers in 2 groups**

**doi:** [**10.1007/s10096-018-3321-8**](https://doi.org/10.1007/s10096-018-3321-8)

Below is text from the results section of this paper describing adverse events

**Adverse events**

No severe adverse events were observed (Table [3](https://pmc.ncbi.nlm.nih.gov/articles/PMC6154122/#Tab3)). Ten adverse events (AE) were reported, but no differences (*p* = 0.40) in the frequency of AEs were detected among the 4 arms (Table [3](https://pmc.ncbi.nlm.nih.gov/articles/PMC6154122/#Tab3)). All AEs were considered not serious. One of the AEs in TRT2 was deemed to be of severe intensity with vulvar itching. Other reported AEs included three that were linked to product use, and the women reported vulvar itching, brown discharges, and vulvovaginal discomfort after administration of the first tablet. However, none of the AEs led to permanent or temporary withdrawal from the study (Table [3](https://pmc.ncbi.nlm.nih.gov/articles/PMC6154122/#Tab3)).

We hope the above examples will persuade the reviewer that the sample size in our Phase I is very similar and comparable to most other similar Phase I trials widely reported and published in the area of microbiome research. Our Phase I trial was approved by Health Canada and the study design, monitoring and data plan was supervised by CIHR Clinical Trials Network (<https://www.hivnet.ubc.ca/>).
